# Role of Human Epicardial Adipose Tissue–Derived miR-92a-3p in Myocardial Redox State

**DOI:** 10.1016/j.jacc.2023.05.031

**Published:** 2023-07-25

**Authors:** Maria Cristina Carena, Ileana Badi, Murray Polkinghorne, Ioannis Akoumianakis, Costas Psarros, Elizabeth Wahome, Christos P. Kotanidis, Nadia Akawi, Alexios S. Antonopoulos, Jagat Chauhan, Rana Sayeed, George Krasopoulos, Vivek Srivastava, Shakil Farid, Nicholas Walcot, Gillian Douglas, Keith M. Channon, Barbara Casadei, Charalambos Antoniades

**Affiliations:** a Cardiovascular Medicine Division, Radcliffe Department of Medicine, University of Oxford, Oxford, United Kingdom; b Department of Genetics and Genomics, College of Medicine and Health Sciences, United Arab Emirates University, Al-Ain, United Arab Emirates; c Cardiothoracic Surgery Department, Oxford University Hospitals NHS Foundation Trust, Oxford, United Kingdom; d Acute Multidisciplinary Imaging and Interventional Centre, Radcliffe Department of Medicine, University of Oxford, Oxford, United Kingdom

**Keywords:** epicardial adipose tissue, microRNAs, myocardial NADPH oxidase activity, myocardial oxidative stress, Wnt5a signaling

## Abstract

**Background:**

Visceral obesity is directly linked to increased cardiovascular risk, including heart failure.

**Objectives:**

This study explored the ability of human epicardial adipose tissue (EAT)-derived microRNAs (miRNAs) to regulate the myocardial redox state and clinical outcomes.

**Methods:**

This study screened for miRNAs expressed and released from human EAT and tested for correlations with the redox state in the adjacent myocardium in paired EAT/atrial biopsy specimens from patients undergoing cardiac surgery. Three miRNAs were then tested for causality in an in vitro model of cardiomyocytes. At a clinical level, causality/directionality were tested using genome-wide association screening, and the underlying mechanisms were explored using human biopsy specimens, as well as overexpression of the candidate miRNAs and their targets in vitro and in vivo using a transgenic mouse model. The final prognostic value of the discovered targets was tested in patients undergoing cardiac surgery, followed up for a median of 8 years.

**Results:**

EAT miR-92a-3p was related to lower oxidative stress in human myocardium, a finding confirmed by using genetic regulators of miR-92a-3p in the human heart and EAT. miR-92a-3p reduced nicotinamide adenine dinucleotide phosphate (NADPH)-oxidase–derived superoxide (O_2_^.–^) by targeting myocardial expression of *WNT5A*, which regulated Rac1-dependent activation of NADPH oxidases. Finally, high miR-92a-3p levels in EAT were independently related with lower risk of adverse cardiovascular events.

**Conclusions:**

EAT-derived miRNAs exert paracrine effects on the human heart. Indeed miR-92a-3p suppresses the wingless-type MMTV integration site family, member 5a/Rac1/NADPH oxidase axis and improves the myocardial redox state. EAT-derived miR-92a-3p is related to improved clinical outcomes and is a rational therapeutic target for the prevention and treatment of obesity-related heart disease.

Cardiovascular disease (CVD) is the leading cause of morbidity and mortality worldwide.^1^ Adipose tissue is now recognized as a crucial regulator of cardiovascular health, mediated by the secretion of several bioactive products (eg, adipocytokines, microvesicles, gaseous messengers) that affect cardiovascular physiology in both an endocrine and paracrine way.^2^^,^^3^ Epicardial adipose tissue (EAT) can affect cardiac physiology in a paracrine way, due to its close anatomical relationship with the myocardium.^2^, ^3^, ^4^ Under physiological conditions, there is a continuous cross-talk between EAT and the myocardium, essential for the maintenance of myocardial health. Although the EAT secretome in chronic diseases may have cardioprotective properties, metabolic dysregulation leads to a shift of the EAT secretome to a pro-oxidant and proinflammatory profile, with detrimental effects on the human heart. The nature of these communication signals between EAT and the heart is poorly understood.

MicroRNAs (miRNAs) are highly conserved, small, single-stranded noncoding RNAs that mainly act as negative posttranscriptional regulators and are rational therapeutic targets in CVD.^5^ Evidence suggests that adipose tissue secretes miRNAs encapsulated into extracellular vesicles that can travel to other organs, affecting their transcriptomic profile.^6^^,^^7^ The role of EAT-derived miRNAs in the regulation of myocardial biology is unclear.

Dysregulated redox signaling plays a role in the pathogenesis of many CVD, and nicotinamide adenine dinucleotide phosphate (NADPH) oxidases (NOXs) are the major enzymatic source of superoxide (O_2_^.–^) in the cardiovascular system.^8^, ^9^, ^10^ All 7 isoforms of NOX identified thus far are multi-transmembrane proteins that are responsible for transporting electrons across biological membranes leading to the reduction of oxygen into O_2_^.–^.^8^ The NOX2-containing NADPH oxidases constitute a major source of O_2_^.–^ in the human myocardium, and their function is dependent on the activation and membrane translocation of the guanosine triphosphatase Rac1 to form the active enzymatic complex. The mechanisms by which EAT affects myocardial redox signaling must still be fully clarified, as that will give new therapeutic options for the prevention and treatment of the cardiac complications of obesity.

The current study investigated the hypothesis that EAT could release miRNAs able to affect the myocardial redox state and hence CVD development. We identified lead miRNAs secreted by human EAT and explored their role in the modulation of myocardial redox signaling, as well as their implication for clinical outcomes in patients with coronary heart disease.

## Methods

### Study Design and Study Population

The study was approved by the Oxfordshire Research Ethics Committee C (REC: 11/SC/0140). The study population consisted of patients undergoing cardiac surgery, all of whom were recruited under the Oxford Heart Vessels and Fat (ox-HVF) program at Oxford University Hospitals NHS Foundation Trust. Exclusion criteria included any inflammatory, neoplastic, renal, or hepatic disease.

Description of the study arms is presented in the Supplemental Methods. The study protocols were in agreement with the Declaration of Helsinki, and all participants had provided written informed consent. The demographic characteristics of these studies are presented in Table 1. Supplemental Figure 1 depicts the study population, goals, and research methodologies of every study arm.

**Table 1:** Demographic Characteristics of Study Participants

	Study Arm 1 (n = 6)	Study Arm 2 (n = 429)	Study Arm 3 (n = 344)	Study Arm 4 (n = 5)	Study Arm 5 (n = 462)
Age, y	67.0 ± 5.5	67.0 ± 0.5	66.0 ± 0.5	72.0 ± 1.5	67.0 ± 0.5
Male	83.3	82.2	86.88	100.0	81.6
CAD	83.3	83.8	99.1	80.0	79.2
Sinus rhythm	100.0	92.0	93.0	100.0	100.0
Persistent AF	0.0	5.9	4.7	0.0	6.3
Postoperative AF	16.7	39.8	38.4	60.0	41.0
Hypertension	50.0	74.7	77.6	100.0	74.0
Hyperlipidemia	100.0	76.1	82.5	100.0	74.8
T2DM	0.0	20.8	23.9	0.0	20.2
Smoking (active/ex)	83.3	63.5	65.9	20.0	62.9
BMI, kg/m^2^	29.80 ± 1.9	28.35 ± 0.2	28.24 ± 0.2	28.06 ± 2.0	28.33 ± 0.2
Waist-to-hip ratio	0.98 ± 0.02	0.98 ± 0.01	0.98 ± 0.01	0.91 ± 0.02	0.97 ± 0.01
hsCRP, mg/L	3.18 ± 0.98	4.23 ± 0.50	4.39 ± 0.59	–	4.19 ± 0.49
Triglycerides, mmol/L	1.32 ± 0.15	1.36 ± 0.04	1.38 ± 0.06	1.09 ± 0.04	1.36 ± 0.05
HDL cholesterol, mmol/L	0.90 ± 0.12	0.92 ± 0.01	0.90 ± 0.01	0.90 ± 0.13	0.92 ± 0.01
LDL cholesterol, mmol/L	2.08 ± 0.32	1.92 ± 0.04	1.80 ± 0.04	1.47 ± 0.18	1.91 ± 0.04
Medications					
ACE inhibitor	33.3	47.8	51.9	80.0	46.0
Antiplatelet	16.7	82.0	90.7	100.0	78.5
ARBs	16.7	14.8	12.8	0.0	15.4
Beta-blockers	50.0	65.8	72.3	100.0	63.1
Statins	83.3	83.1	91.0	100.0	81.3
CCBs	33.3	24.4	25.9	60.0	24.5
Insulin	0.0	7.0	8.2	0.0	6.7
Oral antidiabetic agents	0.0	14.1	16.0	0.0	13.9
Metformin	0.0	12.2	13.7	0.0	11.9
Sulfonylurea	0.0	6.3	7.0	0.0	6.0
DPP4 inhibitors	0.0	1.2	1.5	0.0	1.3
GLP1 analogues	0.0	0.2	0.3	0.0	0.2
SGLT2 inhibitors	0.0	0.0	0.0	0.0	0.2
Thiazolidinediones	0.0	0.2	0.3	0.0	0.2

Values are mean ± SEM or %.

ACE = angiotensin-converting enzyme; AF = atrial fibrillation; ARB = angiotensin receptor blocker; BMI = body mass index; CAD = coronary artery disease; CCB = calcium-channel blocker; DPP4 = dipeptidyl peptidase-4; GLP1 = glucagon-like peptide 1; HDL = high-density lipoprotein; hsCRP = high-sensitivity C-reactive protein; LDL = low-density lipoprotein; SGLT2 = sodium-glucose cotransporter-2; T2DM = type 2 diabetes mellitus.

### Human Tissue Harvesting and Processing

Human EAT (Supplemental Figure 2) and its secretome, as well as myocardial biopsy samples, were harvested and processed as described in the Supplemental Methods.

### MicroRNA Profiling

The screening phase and the confirmation phase of the miRNA profiling are described in the Supplemental Methods.

### Genome-Wide Genetic Screening

Patient genotyping and genome-wide genetic screening are described in the Supplemental Methods. Quantitative trait association tests were conducted using as quantitative phenotypes miR-92a-3p levels in EAT for “EAT-miR-92a-3p” genetic association analysis and miR-92a-3p levels in right atrial appendages (RAAs) for “MYO-miR-92a-3p” genetic association analysis.

### Cell Culture Experiments

The H9c2 cardiomyocytes^11^ culturing and differentiation conditions, as well as protocols for transfection and treatments, are described in the Supplemental Methods and in Supplemental Figure 3. TeloHAEC and human cardiac fibroblast culturing and experiments are also described in the Supplemental Methods.

### Animal Experiments

A doxycycline-inducible wingless-type MMTV integration site family, member 5a (Wnt5a) knock-in mouse model was used to determine the in vivo effects of Wnt5a on myocardial NADPH oxidase activity. C57BL/6, FVB/N Tg(tetO-Wnt5a)17Rva/J (tetO-Wnt5a^+^) and C57BL/6, FVB CAG-rtTA (rtTA^+^) mouse lines have been previously described.^12^ Animal protocols are described in the Supplemental Methods.

### O_2_^.–^ Production Measurements

Myocardial O_2_^.–^ production was measured in the human atrial myocardium or murine hearts using lucigenin (5 μmol/L)-enhanced chemiluminescence, as we have previously described.^4^^,^^13^ O_2_^.–^ was also quantified in cell lysates. Detailed protocols for O_2_^.–^ quantification are presented in the Supplemental Methods.

### RNA Isolation and Quantitative Reverse Transcription Polymerase Chain Reaction

RNA isolation and quantitative reverse transcription polymerase chain reaction methods are described in the Supplemental Methods.

### Rac1 Activation

Rac1 activation was assessed by using an active Rac1 detection kit (Cell Signaling Technology). Details are provided in the Supplemental Methods.

### Rac1 Membrane Translocation

Membrane translocation of Rac1 was estimated by differential centrifugation of cell lysates or tissue homogenates to isolate membrane proteins as previously described.^12^ Details are provided in the Supplemental Methods.

### Western Blot Analysis

Western immunoblot analysis was performed on myocardium homogenates and cell lysates (Supplemental Methods).

### Statistical analysis

Continuous variables were tested for normal distribution by using the Kolmogorov-Smirnov test. Non-normally distributed variables are presented as median (25th-75th percentile) and whiskers (from 10th to 90th percentile) for n ≥ 30. In experiments with a small sample size, variables are presented as scatterplots with median values.^14^^,^^15^ Statistical tests used for correlation analyses, comparisons between groups, and regression analyses are detailed in the Supplemental Methods.

All statistical tests were 2-tailed and were performed by using SPSS version 20.0 (IBM SPSS Statistics, IBM Corporation). A value of *P* < 0.05 was considered statistically significant.

## Results

### Human EAT Releases miR-92a-3p That Affects Myocardial Superoxide Production

**Figure 1:**
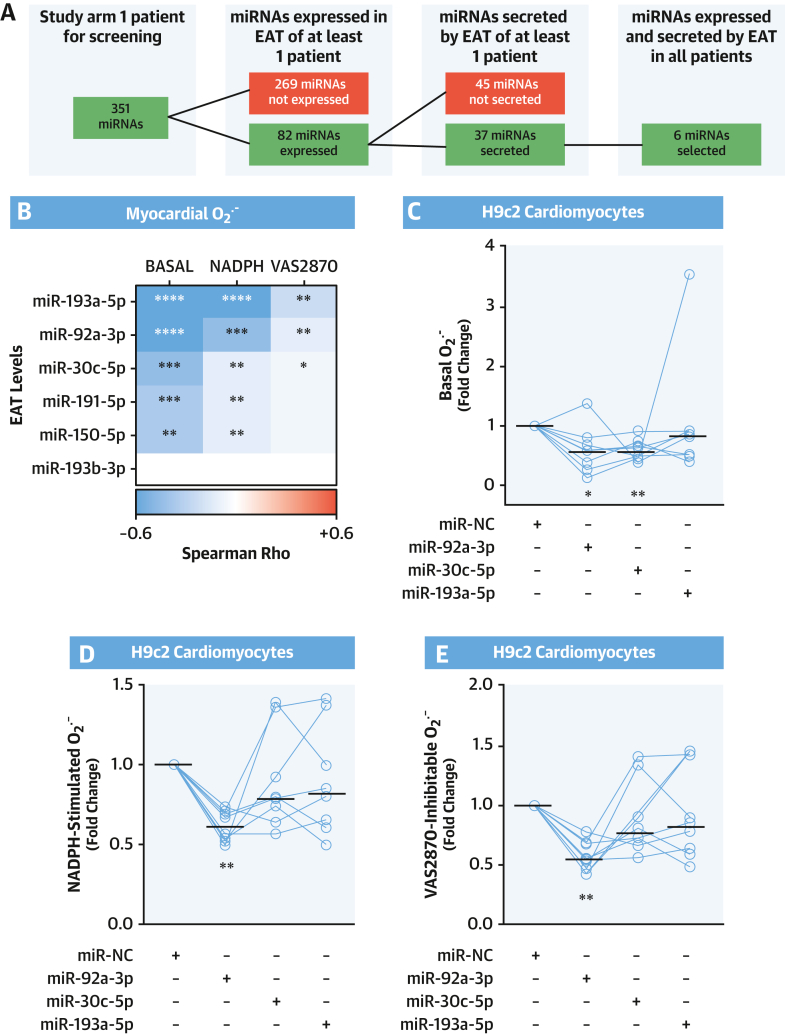
EAT-Derived miR-92a-3p and Myocardial O_2_^.–^ Production **(A)** Flowchart showing the screening process to identify microRNAs (miRNAs) expressed and released by epicardial adipose tissue (EAT). **(B)** Heatmap showing the Spearman correlation coefficient between the EAT levels of the 6 identified miRNAs and the basal, nicotinamide adenine dinucleotide phosphate (NADPH)-stimulated, and VAS2870-inhibitable superoxide (O_2_^.–^) production in the human myocardium (n = 56). ∗*P* < 0.05, ∗∗*P* < 0.01, ∗∗∗*P* < 0.001, ∗∗∗∗*P* < 0.0001. **(C to E)** Basal, NADPH-stimulated, and VAS2870-inhibtable O_2_^.–^ generation in differentiated H9c2 cardiomyocytes transfected with an miRNA mimic negative control (miR-NC) or miR-30c-5p, miR-92a-3p, and miR-193a-5p mimic (n = 8). Lines on scatterplots represent medians. ∗*P* < 0.05, ∗∗*P* < 0.01 vs control by Wilcoxon signed-rank test.

A microarray analysis was performed on EAT and EAT-derived supernatants from patients who underwent cardiac surgery at Oxford University Hospitals (study arm 1) (Supplemental Figure 1) to identify microRNAs expressed and released by EAT that might regulate gene expression in the adjacent myocardium. Patient characteristics are summarized in Table 1. Of the 351 tested microRNAs, only 37 were expressed in EAT, and only 6 of them (miR-30c-5p, miR-92a-3p, miR-150-5p, miR-191-5p, miR-193a-5p, and miR-193b-3p) were consistently detected also in the secretome of all EAT samples collected at the end of the 4-hour ex vivo culture, and included into the screening (Figure 1A, Supplemental Figure 4A). Expression of these 6 candidate miRNAs was next confirmed in EAT samples from 206 patients (study arm 2) (Supplemental Figure 1, Table 1) by quantitative reverse transcription polymerase chain reaction (Supplemental Figure 4B), confirming their biological relevance in human EAT.

We then investigated whether expression of the microRNAs expressed and secreted by the human EAT was associated with myocardial redox state, measured in myocardial biopsy samples from RAAs of the same patients in whom the miRNAs levels in EAT were measured. Indeed, miR-30c-5p, miR-92a-3p, and miR-193a-5p expression levels in EAT were negatively correlated with basal myocardial O_2_^.–^ production, as detected by lucigenin-enhanced chemiluminescence (Figure 1B, Supplemental Figure 5). Furthermore, these miRNAs were negatively correlated with myocardial NADPH-stimulated O_2_^.–^ and the level of O_2_^.–^ inhibitable by VAS2870, a specific pan-NOX inhibitor. In contrast, the levels of miR-150-5p, miR-191-5p, and miR-193b-3p were not significantly correlated with myocardial NADPH oxidase-dependent O_2_^.–^ production. These findings identify a possible role for EAT-derived microRNAs in the regulation of myocardial redox state in humans via effects on NADPH oxidase activity.

We next performed in vitro mechanistic experiments to explore the causal role of miR-30c-5p, miR-92a-3p, and miR-193a-5p in regulating myocardial NADPH oxidase activity. Differentiated H9c2 cardiomyocytes (Supplemental Figure 3) were transfected with locked nucleic acid–based mimics of miR-30c-5p, miR-92a-3p, or miR-193a-5p. Transfection of miR-30c-5p and miR-92a-3p reduced basal O_2_^.–^ generation (Figure 1C). However, only miR-92a-3p significantly reduced cardiomyocyte NADPH-stimulated and VAS2870-inhibitable O_2_^.–^ production (Figures 1D and 1E). Considering that NADPH-stimulated and VAS2870-inhibitable O_2_^.–^ generation are the most specific readouts of NADPH oxidase activity, these findings suggest that miR-92a-3p has the most consistent effect on NADPH oxidase activity. Interestingly, we found that miR-92a-3p could reduce NADPH oxidase–dependent O_2_^.–^ generation only in differentiated H9c2 cardiomyocytes and not in other cardiovascular cell types such as endothelial cells or cardiac fibroblasts (Supplemental Figure 6).

**Figure 2:**
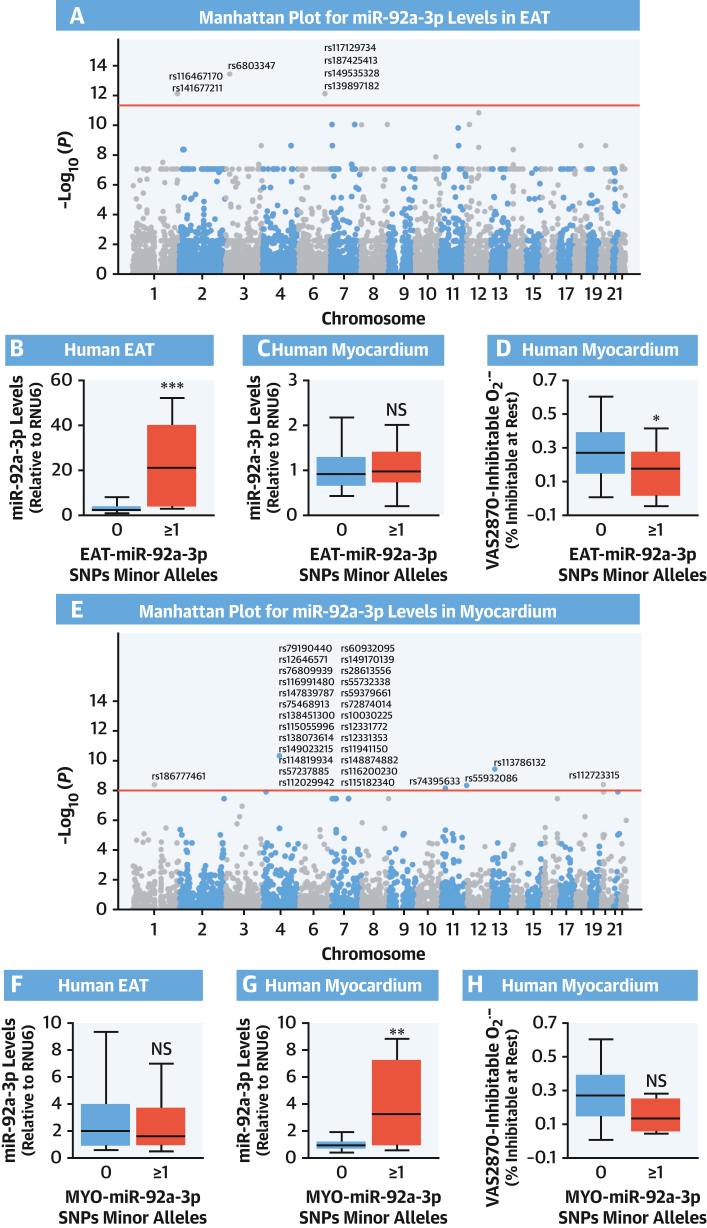
SNPs Affecting EAT miR-92a-3p and Myocardial Redox State Manhattan plots representing single-nucleotide polymorphisms (SNPs) associated with miR-92a-3p levels in EAT **(A)** and myocardium **(E)**. Seven SNPs were significantly associated with high miR-92a-3p levels in EAT (EAT-miR-92a-3p) and 31 with high levels of miR-92a-3p in the myocardium (MYO-miR-92a-3p). The presence of any SNP from EAT-miR-92a-3p was associated with higher miR-92a-3p levels only in EAT, not in the myocardium (**B**, n = 149; **C**, n = 265), whereas the presence of any MYO-miR-92a-3p SNP was associated with higher miR-92a-3p levels only in the myocardium, not in EAT (**G**, n = 265; **F**, n = 149). The presence of any EAT-miR-92a-3p SNP led to a statistically significant reduction of myocardial superoxide production (**D** and **H**, n = 196). In **B to D** and **F to H**, data are presented as median (25th-75th percentile). ∗*P* < 0.05, ∗∗*P* < 0.01, ∗∗∗*P* < 0.001 by the Mann-Whitney *U* test. NS = not significant; other abbreviations as in Figure 1.

To support our hypothesis that EAT-derived miR-92a-3p is causally associated with myocardial redox state in humans, we performed genetic screening (using the UK Biobank Genome-Wide Association Studies [GWAS] array, study arm 3) to identify the leading single-nucleotide polymorphisms (SNPs) (ie, reaching genome-wide significance as stated) associated with the levels of miR-92a-3p in EAT (EAT-miR-92a-3p) or myocardium (MYO-miR-92a-3p) (Figures 2A and 2E). Minor alleles associated with EAT-miR-92a-3p were related to high miR-92a-3p levels in EAT but not in the myocardium (Figures 2B and 2C), whereas minor alleles associated with MYO-miR92a were associated with miR-92a-3p levels only in the myocardium but not in EAT (Figures 2F and 2G), indicating allele- and tissue-specific regulation of cardiac miR-92a-3p levels. Notably, only minor alleles associated with high miR-92a-3p levels in EAT were related to lower myocardial O_2_^.–^ production (Figure 2D), whereas the trends observed for the alleles driving MYO-miR-92a-3p were not statistically significant (Figure 2H).

### miR-92a-3p Modulates O_2_^.–^ Production in Cardiomyocytes Through Akt

**Figure 3:**
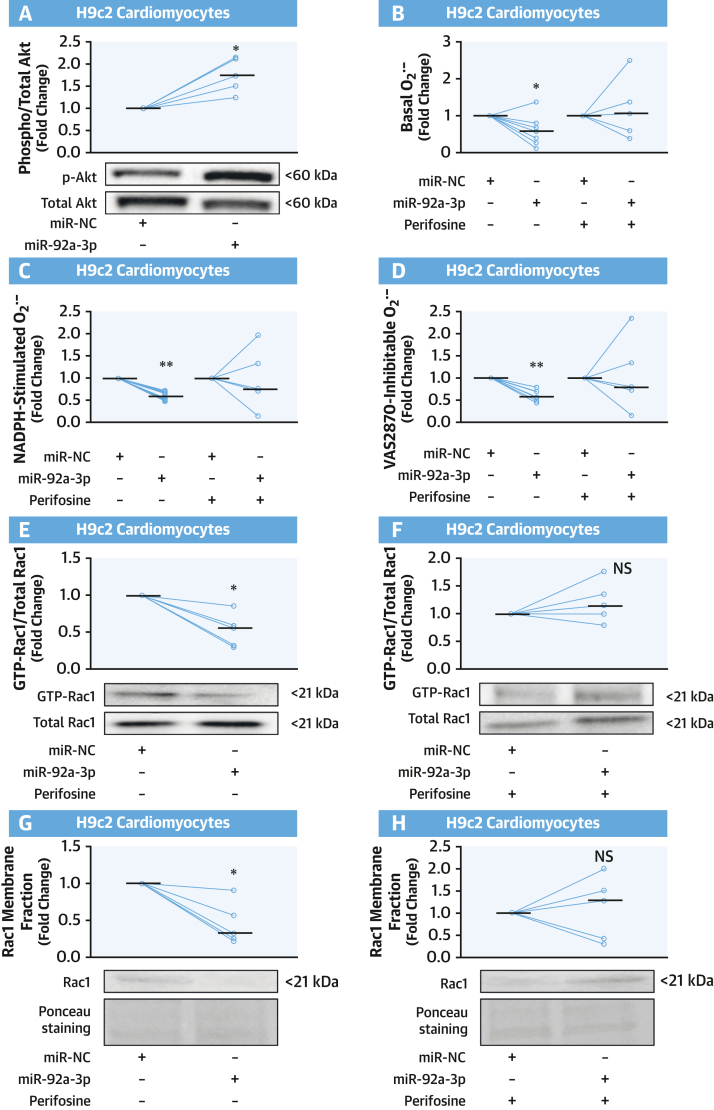
miR-92a-3p Modulates O_2_^.–^ Production in Cardiomyocytes Through Akt **(A)** Transfection of differentiated H9c2 cardiomyocytes with miR-92a-3p mimic increased the phosphorylation of Akt protein kinase at Ser473 compared with an miR-NC (n = 5). **(B to D)** miR-92a-3p expression in differentiated H9c2 cardiomyocytes suppressed NADPH-stimulated and VAS2870-inhibtable O_2_^.–^ compared with miR-NC, an effect abolished in the presence of the Akt inhibitor perifosine (n = 5-8). **(E to H)** Fold change of activated Rac1 (measured as ratio of guanosine triphosphate [GTP]-Rac1:total Rac1) and of Rac1 membrane translocation in H9c2 cardiomyocytes transfected with either miR-NC or a miR-92a-3p mimic ± Akt inhibitor perifosine (n = 5). Lines on scatterplots represent medians. ∗*P* < 0.05, ∗∗*P* < 0.01 vs controls by Wilcoxon signed-rank test. Abbreviations as in Figure 1.

We have previously shown that NADPH oxidase activity can be decreased by Akt-mediated inhibition of Rac1 activation.^16^ Therefore, we investigated whether the observed inhibitory effects of miR-92a-3p on NADPH oxidase activity were mediated via the Akt/Rac1 axis. We first evaluated whether miR-92a-3p could affect the activation of Akt, as assessed by its phosphorylation at Ser473. miR-92a-3p mimic transfection in cardiomyocytes increased phospho-Akt levels (Figure 3A). Treatment with perifosine, a known inhibitor of Akt phosphorylation at Ser473 and Thr308 residues,^17^ inhibited the miR-92a-3p–mediated decrease in O_2_^.–^ generation in this cell type (Figures 3B to 3D). As expected, miR-92a-3p reduced Rac1 guanosine triphosphate (GTP) activation and Rac1 membrane translocation, and this effect was reversed by perifosine (Figures 3E to 3H). These results suggest that the inhibitory effect of miR-92a-3p on NADPH oxidase activity occurs via Akt-mediated inhibition of Rac1 activation.

### miR-92a-3p Regulates Wnt5a Protein Levels in Cardiomyocytes

**Figure 4:**
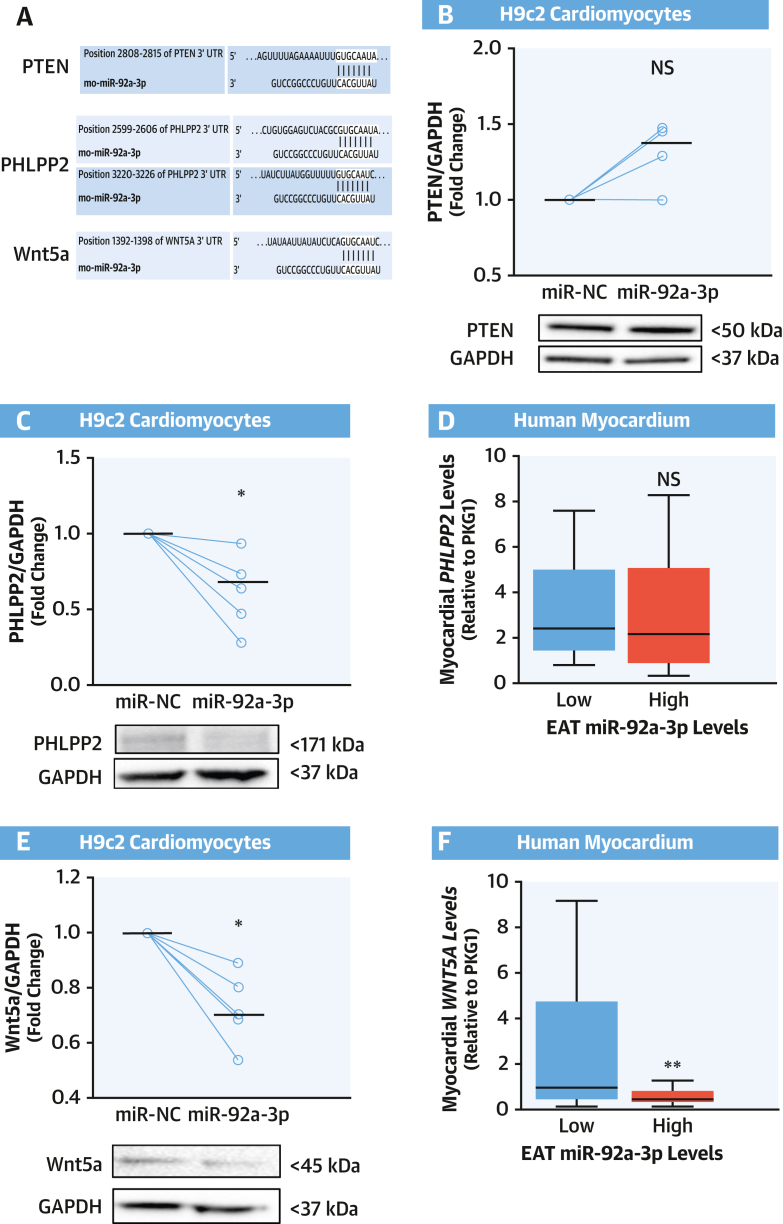
miR-92a-3p Downregulates Protein Levels of Wnt5a in Cardiomyocytes **(A)** Prediction of miR-92a-3p binding to phosphatase and tensin homolog (PTEN), PH domain and leucine-rich repeat protein phosphatase 2 (PHLPP2), and wingless-type MMTV integration site family, member 5a (Wnt5a) 3′-untranslated regions (UTRs) assessed by using TargetScan software version 7.2 (TargetScan). Transfection of H9c2 cardiomyocytes with miR-92a-3p mimic resulted in the reduction of protein levels of PHLPP2 **(C)** and Wnt5a **(E)**, but not PTEN **(B),** compared with the miR-NC (n = 4-6). **(D and F)** Patients with high levels (above the median) of miR-92a-3p in EAT had lower myocardial expression of *WNT5A* (but not *PPLPP2*) (n = 71). Data are presented as median (25th-75th percentile). Lines on scatterplots represent medians. ∗*P* < 0.05 vs control by Wilcoxon signed-rank test. ∗∗*P* < 0.01 by Mann-Whitney *U* test. GAPDH = glyceraldehyde 3-phosphate dehydrogenase; other abbreviations as in Figures 1 and 3.

To understand how miR-92a-3p affects Rac1 activation, we performed a bioinformatics analysis to identify putative targets of miR-92a-3p that are known to also interact with Rac1 (Supplemental Figure 7). In accordance with our in vitro results, this analysis revealed a subset of molecules known to play a role in Akt signaling.^18^ Thus, we next explored whether miR-92a-3p may modulate putative targets (phosphatase and tensin homolog [PTEN], PH domain and leucine-rich repeat protein phosphatase 2 [PHLPP2], and Wnt5a) (Figure 4A) that are known to decrease Akt phosphorylation.^18^, ^19^, ^20^ Although PTEN has been already linked to Akt and the Rac1 pathway, and is one of the most investigated miR-92a-3p targets,^18^^,^^21^^,^^22^ its protein levels were unchanged in cardiomyocytes transfected with miR-92a-3p (Figure 4B), whereas both PHLPP2 and Wnt5a were downregulated by miR-92a-3p (Figures 4C and 4E).

To understand the relevance of these findings in humans, we assessed miR-92a-3p levels in EAT and the expression levels of these 2 potential miR-92a-3p targets in myocardial samples from patients of study arm 2. Patients with high miR-92a-3p levels in EAT exhibited lower myocardial levels of *WNT5A,* whereas there were no significant differences in *PHLPP2* expression in the myocardium of patients with low vs high EAT miR-92a-3p levels (Figures 4D and 4F). Collectively, these data suggest that Wnt5a could be a target of miR-92a-3p with a role in myocardial Rac1-dependent O_2_^.–^ production.

### Wnt5a Increases Myocardial O_2_^.–^ Generation Through Rac1-Mediated Activation of NADPH Oxidases

**Figure 5:**
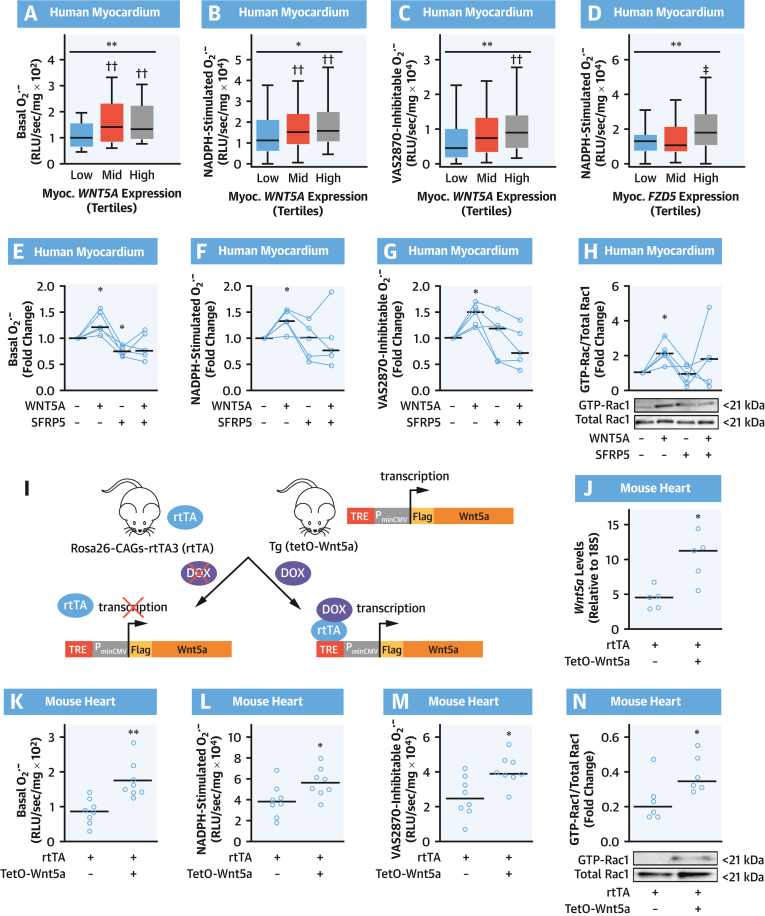
WNT5A Increases O_2_^.–^ Generation in Human and Murine Myocardium **(A to D)** Basal, NADPH-stimulated, and VAS2870-inhibtable O_2_^.–^ production in human myocardium according to tertiles of *WNT5A* or *FZD5* expression in myocardium (n = 189 in **A to C**; n = 181 in **D**). Data are presented as median (25th-75th percentile). ∗*P* < 0.05, ∗∗*P* < 0.01 by Kruskal-Wallis test; ††*P* < 0.01 vs low tertile by Dunn’s test corrected for multiple tests. **(E to H)** O_2_^.–^ production and fold change of activated Rac1 in human myocardium in presence/absence of WNT5A and SFRP5 (n = 5). ∗*P* < 0.05 vs control by Wilcoxon signed paired rank test. **(I)** Breeding scheme for inducible expression of FLAG-tagged Wnt5a. **(J)** Doxycycline (DOX) treatment induces marked Wnt5a overexpression in Wnt5a^+^/rtTA^+^ hearts (n = 5). O_2_^.–^ production **(K to M)** and Rac1 activation **(N)** in hearts of DOX-treated mice (n = 6-8 per group). Lines on scatterplots represent median values. ∗*P* <0.05, ∗∗*P* < 0.01 vs control by unpaired *t* test. Myoc = myocardial; P_minCMV_ = promoter; RLU = relative light unit; rtTA = reverse tetracycline-controlled transactivator; TRE = tetracycline-response element; other abbreviations as in Figures 1 and 4.

We next explored the role of Wnt5a in regulating myocardial redox state. First, we looked at myocardial O_2_^.–^ production and NADPH oxidase activity and *WNT5A* expression in the human myocardium. Myocardial WNT5A transcript levels were significantly increased in patients with increased basal (Figure 5A), NADPH-stimulated (Figure 5B), and VAS2870-inhibitable (Figure 5C) generation of O_2_^.–^ in the myocardium. In agreement with these findings, the expression of Wnt5a receptors Frizzled class receptor 2 (FZD2) and Frizzled class receptor 5 (FZD5) in the human myocardium was increased in patients with high myocardial NADPH-stimulated O_2_^.–^ (Figure 5D, Supplemental Figure 8). These findings are consistent with the presence of an interaction between Wnt5a signaling and myocardial NADPH oxidase activity in humans. Wnt signaling is mediated by membrane receptors, including Fzd2, Fzd5, receptor tyrosine kinase–like orphan receptors 1 and 2 (Ror1-2), and receptor-like tyrosine kinase (Ryk),^23^ and it is regulated by secreted frizzled related proteins (Sfrp), which act as decoy receptors, reducing the bioavailability of Wnt ligands.^23^

**Figure 6:**
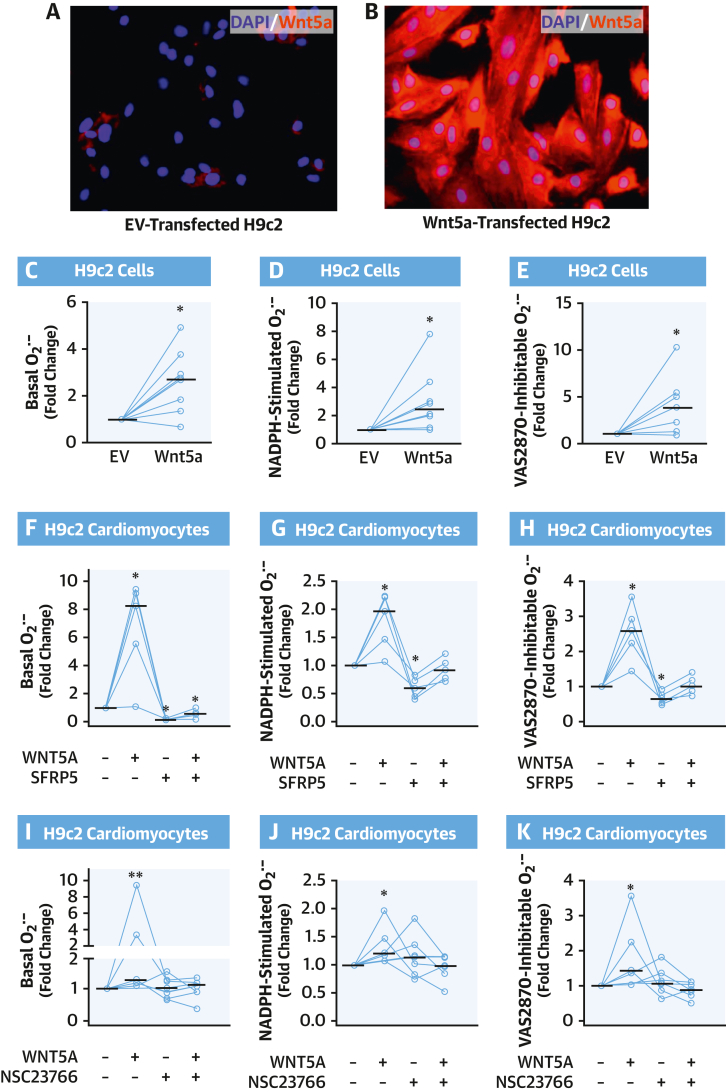
Wnt5a Increases O_2_^.–^ Generation in Cardiomyocytes Through Rac1-Mediated NOX Activity **(A and B)** Overexpression of FLAG-tagged Wnt5a in H9c2 cells was evaluated by immunofluorescence with anti-FLAG antibody **(red)** and compared with H9c2 cells transfected with an empty vector (EV); nuclei were stained with 4′,6-diamidino-2-phenylindole (DAPI) **(blue)**. **(C to E)** Basal, NADPH-stimulated, and VAS2870-inhibtable O_2_^.–^ in H9c2 cells overexpressing or not FLAG-tagged Wnt5a (n = 8). **(F to H)** O_2_^.–^ production in H9c2 cardiomyocytes in the presence/absence of WNT5A and SFRP5 (n = 5). **(I to K)** O_2_^.–^ production in H9c2 cardiomyocytes treated with or without WNT5A and NSC23766, a specific RAC1 inhibitor (n = 5-7). Lines represent medians. ∗*P* < 0.05, ∗∗*P* < 0.01 vs control by Wilcoxon signed-rank test. Abbreviations as in Figures 1 and 4.

To explore whether the association between Wnt5a and myocardial NADPH oxidase activity was causal, we performed ex vivo incubations of human myocardial tissue from patients of study arm 4, exposing RAA samples to human recombinant WNT5A and/or its decoy receptor secreted frizzled–related protein 5 (SFRP5). WNT5A induced a significant increase in myocardial NADPH oxidase–dependent O_2_^.–^ generation; this effect was reversed by SFRP5 (Figures 5E to 5G). Furthermore, WNT5A directly increased GTP activation of Rac1 (Figure 5H). To further support causality and to confirm the molecular mechanisms through which WNT5A could increase myocardial O_2_^.–^ production, additional in vivo experiments were performed by using a doxycycline-inducible Wnt5a-overexpressing mouse model (Wnt5a^+^/rtTA^+^ mice) (Figure 5I).^12^ Treatment of Wnt5a^+^/rtTA^+^ mice with doxycycline induced marked overexpression of Wnt5a in multiple tissues, including the heart, compared with doxycycline-treated Wnt5a^–^/rtTA^+^ littermate controls (Figure 5J).^12^ In accordance with our ex vivo experiments, we found that doxycycline-treated Wnt5a^+^/rtTA^+^ mice exhibited elevated basal, NADPH-stimulated, and VAS2870-inhibitable O_2_^.–^ generation (Figures 5K to 5M) as well as increased GTP-Rac1 activation (Figure 5N) in myocardial tissue compared with doxycycline-treated Wnt5a^–^/rtTA^+^ littermates. Similarly, Wnt5a overexpression in H9c2 cells induced increased NADPH oxidase-dependent O_2_^.–^ generation and GTP-Rac1 activation (Figures 6A to 6E, Supplemental Figure 9). Identical results were obtained by treating cardiomyocytes with human WNT5A recombinant protein (Figures 6F to 6H). Furthermore, SFRP5 could reverse the WNT5A-mediated increase in O_2_^.–^production.

Finally, to test the notion that WNT5A acts through Rac1 to induce NADPH oxidase-dependent O_2_^.–^ generation, we preincubated cardiomyocytes with NSC23766, a Rac1 inhibitor, before exposing them to WNT5A. NSC23766 abolished the ability of WNT5A to increase basal (Figure 6I), NADPH-stimulated (Figure 6J), or VAS2870-inhibitable (Figure 6K) O_2_^.–^ production. Taken together, these results show that WNT5A increases myocardial O_2_^.–^ generation through Rac1-mediated NADPH oxidase activity, contrasting the observed effects of miR-92a-3p on myocardial O_2_^.–^ generation.

### Clinical Implications of EAT-Derived miR-92a-3p and Myocardial WNT5A

**Figure 7:**
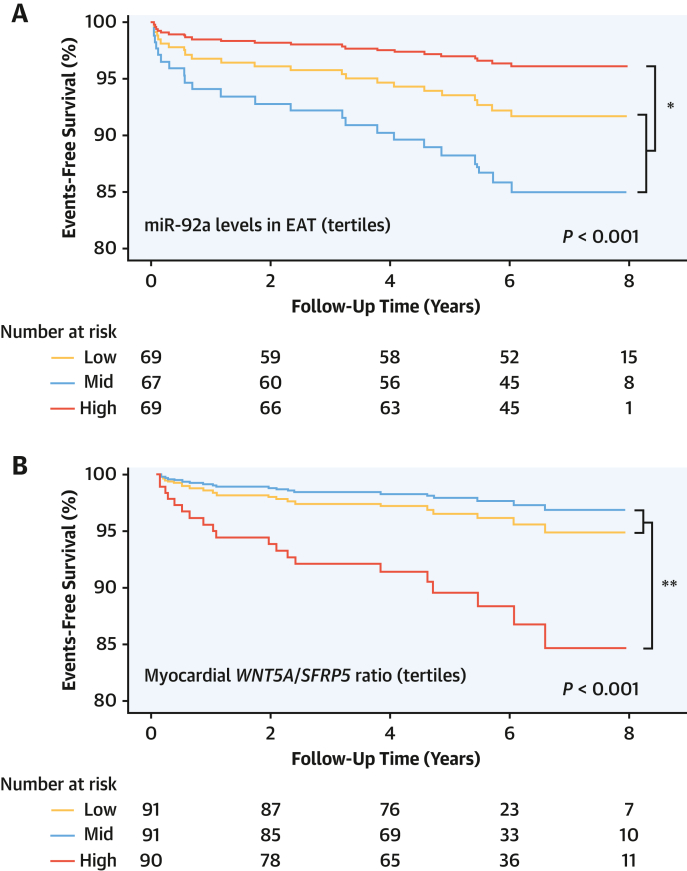
Association of EAT miR-92a-3p Levels With Risk of Cardiovascular Events Prognostic value of epicardial adipose tissue (EAT) miR-92a-3p levels **(A)** and myocardial *WNT5A/SFRP5* levels **(B)** for the composite outcome of cardiac mortality, nonfatal myocardial infarction, and nonfatal stroke. The *P* values are calculated from Cox regression after adjusting for age, sex, hypertension, body mass index, and diabetes. ∗*P* < 0.05, ∗∗*P* < 0.01 for highest tertile vs pooled mid and lowest tertiles. Other abbreviations as in Figures 1 and 4.

To explore whether these newly described effects of EAT-derived miR-92a-3p on myocardial redox state would translate to clinical phenotypes associated with cardiovascular outcomes, we followed up the population of study arm 5 for up to 8 years, through nationwide data collection via the NHS Digital coding system, to ascertain adverse clinical events. Patients with higher miR-92a-3p levels in EAT at baseline had a significantly lower risk for the composite outcome of cardiac mortality, nonfatal myocardial infarction (MI), and nonfatal cardioembolic stroke after adjusting for age, sex, hypertension, body mass index, and diabetes (HR: 0.328; 95% CI: 0.11-0.98; *P* = 0.046 for the highest tertile of miR-92a-3p levels in EAT vs the rest) (Figure 7A). In line with our ex vivo findings, patients with higher *WNT5A/SFRP5* myocardial expression had a significantly higher risk for the same composite outcome of cardiac mortality, nonfatal MI, and nonfatal cardioembolic stroke (HR: 3.941; 95% CI: 1.607-9.661; *P* = 0.003 for highest tertile of *WNT5A/SFRP5* myocardial expression vs the rest) (Figure 7B). Patients with sinus rhythm at the time of surgery whose EAT miR-92a-3p levels were within the highest 2 tertiles had significantly lower risk of developing postoperative atrial fibrillation compared with those at the lowest tertile (HR: 0.56; 95% CI: 0.32-0.98; *P* = 0.043) after adjusting for age, sex, hypertension, smoking, hypercholesterolemia, body mass index, ethnicity, and ejection fraction.

We also compared the demographic characteristics and other clinical factors among patients grouped according to tertiles of EAT miR-92a-3p levels. Although there was no significant difference in most of the risk factors between groups, patients with high miR-92a-3p levels were more likely to be taking antiplatelet medication or to have hypercholesterolemia (Supplemental Table 1).

## Discussion

Adipose tissue communicates with the cardiovascular system via the secretion of endocrine and paracrine signals, which include adipokines/adipocytokines,^3^^,^^24^ lipid species,^25^ microRNAs,^26^ and other molecules.^12^ Although, under physiological conditions, adipose tissue supports cardiovascular health, its secretome in the presence of metabolic dysregulation becomes detrimental to the cardiovascular system.^2^^,^^3^ EAT also exerts direct paracrine effects on the adjacent myocardium,^4^ but it is unclear whether it secretes microRNAs with any paracrine regulatory role in the human heart. We now identify for the first time a range of microRNAs expressed and secreted by EAT, which seem to have a regulatory role on myocardial redox state in humans. The expression of one of these microRNAs in the human EAT, miR-92a-3p, was found to be inversely correlated with myocardial redox state, and particularly the generation of O_2_^.–^ from NADPH oxidases in the human heart. Causality was documented by using a Mendelian randomization approach in humans, as well as by showing that miR-92a-3p decreases NADPH oxidase–derived O_2_^.–^ generation in cardiomyocytes in vitro. We then identified Wnt5a as a possible mediator of miR-92a-3p effects on myocardial redox state. Indeed, miR-92a-3p reduces the expression of Wnt5a through direct binding to its 3′-untranslated region.^20^ Accordingly, Wnt5a protein levels were downregulated by miR-92a-3p in cardiomyocytes, and myocardial Wnt5a levels were also inversely correlated with miR-92a-3p levels in the adjacent EAT in our population. Ex vivo, in vivo, and in vitro experiments also showed that Wnt5a increases myocardial O_2_^.–^ production through Rac1-mediated NADPH oxidase activity (Central Illustration). The clinical importance of our findings is highlighted by the association of EAT miR-92a-3p levels with a lower risk of major adverse cardiovascular events (MACE), as well as postoperative atrial fibrillation, and the positive association of MACE risk with *WNT5A* expression in patients with advanced atherosclerosis. These findings suggest a threshold effect when miR-92a-3p EAT levels exceed the lowest tertile that leads to higher *WNT5A* expression levels in the myocardium, which may drive cardiovascular events.

**Central Illustration:**
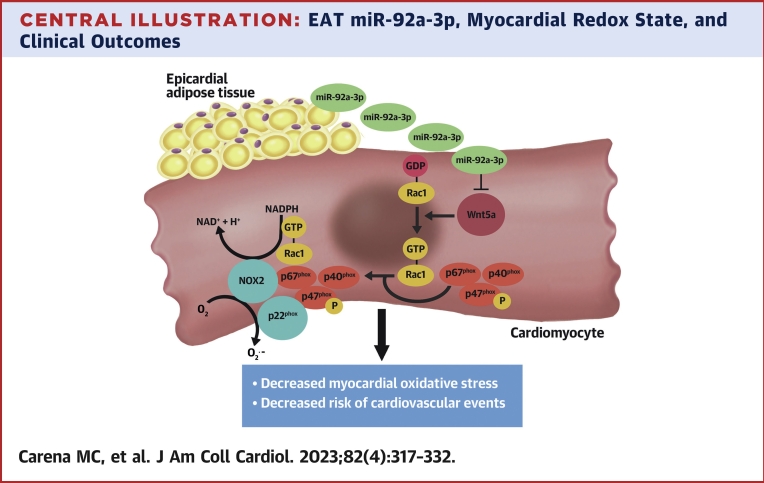
EAT miR-92a-3p, Myocardial Redox State, and Clinical Outcomes Epicardial adipose tissue (EAT) expresses and releases miR-92a-3p that decreases superoxide production in cardiomyocytes possibly by targeting wingless-type MMTV integration site family, member 5a (Wnt5a). Wnt5a induces guanosine triphosphate (GTP) activation of Rac1 that translocates to the cell membrane together with other cytosolic regulatory subunits to form an active enzymatic complex containing nicotinamide adenine dinucleotide phosphate (NADPH) oxidases (eg, NOX2), which catalyzes the production of superoxide (O_2_^.–^). High EAT miR-92a-3p is associated with lower cardiovascular risk.

EAT largely affects myocardial biology as it secretes different molecules that are involved in several cardiac biological processes, such as hypertrophy, redox balance, contractility, inflammation, and fibrosis.^3^ Although it is established that the expression of miRNAs in EAT is altered in patients with CVD or conditions increasing the risk of CVD, such as obesity and type 2 diabetes mellitus, the underlying mechanisms of action of EAT-secreted miRNAs on myocardial biology are unclear.^27^, ^28^, ^29^, ^30^, ^31^ This is the first study that describes a miRNA released by EAT that can act as a modulator of myocardial redox signaling.

Using human EAT secretome profiling, we identified miR-92a-3p as being expressed and released by EAT. miR-92a-3p levels in EAT negatively correlated with NADPH oxidase–dependent O_2_^.–^ production in the human myocardium, and the causal role of this miRNA in redox signaling modulation was proven as it could decrease NOX activity in the current in vitro model of cardiomyocytes.

miR-92a-3p belongs to the miR-17-92 cluster and, in addition to playing a role in tumor biology, it is a key regulator of angiogenesis after MI.^32^, ^33^, ^34^, ^35^, ^36^ However, its role to date in myocardial redox signaling regulation has been completely unexplored. For the first time, we show that miR-92a-3p decreases cardiomyocyte O_2_^.–^ generation mediated by Rac1-dependent NADPH oxidase activity; this effect is specific to cardiomyocytes, while opposite effects are observed in other cell types (eg, endothelial cells). We also proved that this novel function of miR-92a-3p is mediated by Akt and possibly by downregulation of its target Wnt5a. The Akt inhibitor perifosine could indeed counteract the miRNA-mediated decrease in Rac1 activation/membrane translocation and consequent O_2_^.–^ production, and miR-92a-3p transfection in cardiomyocytes downregulated protein levels of Wnt5a. The concept that EAT-derived miR-92a-3p could regulate myocardial Wnt5a is supported by a negative correlation between miR-92a-3p levels in EAT and myocardial *WNT5A*. Furthermore, it has been shown that miR-92a-3p directly binds to the 3′-untranslated region of Wnt5a to exert its extracellular repressive function; for example, Mao et al^20^ showed that miR-92a-3p secreted by mesenchymal stem cells in exosomes can regulate Wnt5a protein levels in chondrocytes. Most importantly, Wnt5a has opposite effects on myocardial Rac1-dependent NADPH oxidase activity compared with miR-92a-3p. This novel Wnt5a function in myocardial biology was proven by means of ex vivo, in vivo, and in vitro experiments supporting the notion that miR-92a-3p could modulate myocardial redox signaling through Wnt5a downregulation. The opposite effects of EAT-derived miR-92a-3p and myocardial Wnt5a on redox signaling in the human myocardium translate also in clinical phenotypes as levels of this miRNA in EAT and levels of *WNT5A/SFRP5* in the myocardium are negatively and positively associated, respectively, with risk of MACE.

### Study Limitations

Although this study identified EAT-derived miR-92a-3p and its myocardial targets (Wnt5a/Rac1/NADPH oxidase activity) as predictors of cardiac outcomes, it is unclear how we could intervene pharmacologically. It is also unclear whether remote adipose tissue depots (eg, visceral or subcutaneous adipose tissue) could also secrete miR-92a-3p that might exert endocrine effects on the human heart. Furthermore, miRNA-92a-3p from human EAT may affect the myocardium in a paracrine way, but a definitive causal relationship has not been shown, and this is a limitation of the study. Moreover, in this study, we used differentiated H9c2 cardiomyocytes instead of primary human cardiomyocytes as an in vitro model for the mechanistic experiments. Finally, the paired RAA and the EAT from the atrioventricular groove were in anatomical proximity but not directly adjacent to each other; however, samples from the right atrioventricular groove have similar miR-92a-3p expression compared with samples directly adjacent to the RAA (Supplemental Figure 2).

## Conclusions

The current study shows that high EAT-derived miR-92a-3p is associated with improved clinical cardiovascular outcomes and that this microRNA decreases myocardial oxidative stress, possibly by targeting the Wnt5a/Rac1/NADPH oxidase axis (Central Illustration).

Perspectives **COMPETENCY IN MEDICAL KNOWLEDGE:** EAT-derived microRNAs exert paracrine effects on human myocardium, miR-92a-3p improves regulation of the human myocardial redox state, and increased miR-92a-3p levels in EAT reduce cardiovascular risk.

**TRANSLATIONAL OUTLOOK:** Further research is needed to clarify the role of miR-92a-3p in various cardiac cell types.

## Funding Support and Author Disclosures

This study was supported by a Marie Skłodowska-Curie Early Stage Researcher fellowship to Dr Carena; the CATCH ME (Characterizing Atrial fibrillation by Translating its Causes into Health Modifiers in the Elderly) consortium (grant number 633196); the British Heart Foundation (FS/16/15/32047, RG/F/21/110040 and CH/F/21/90009 to Dr Antoniades; CH/16/1/32013 to Dr Channon; CH/12/3/29609 to Dr Casadei); Oxford BHF Centre of Research Excellence RE/18/3/34214, the Oxford NIHR Biomedical Research Centre, the National Institute for Health Research Oxford Biomedical Research Centre, and the Novo Nordisk Foundation (NNF15CC0018486) to Dr Antoniades. Dr Antoniades has had consultancy agreements with Mitsubishi Tanabe and Silence Therapeutics; has received grants from Sanofi and Novo Nordisk; is the Chair of the British Atherosclerosis Society; and has received honoraria from Amarin and Covance. Drs Antoniades and Channon are founders, shareholders, and directors of Caristo Diagnostics. Dr Casadei is the past president of the European Society of Cardiology. All other authors have reported that they have no relationships relevant to the contents of this paper to disclose.
