# Supplementary material for: Role of Human Epicardial Adipose Tissue–Derived miR-92a-3p in Myocardial Redox State
Source: J Am Coll Cardiol. 2023 Jul 25;82(4):317–32. doi: 10.1016/j.jacc.2023.05.031 (PMC10368522; doi:10.1016/j.jacc.2023.05.031)
Supplement: Supplemental Material [file mmc1.docx]

-SUPPLEMENTAL MATERIAL-

Role of Human Epicardial Adipose Tissue–Derived miR92a-3p in Myocardial Redox State

**Brief title:** Myocardial Effects of Adipose Tissue miRNA

**Total Word Count:** 4969

Maria Cristina Carena, PhD^a,*^, Ileana Badi, PhD^a,*^, Murray Polkinghorne, MBChB, MPhil^a^, Ioannis Akoumianakis, MD, DPhil^a^, Costas Psarros, PhD^a^, Elizabeth Wahome^a^, Christos P Kotanidis, MD, DPhil^a^, Nadia Akawi, PhD^a,b^, Alexios S Antonopoulos, MD, PhD^a^, Jagat Chauhan, PhD^a^, Rana Sayeed, MD, PhD^c^, George Krasopoulos, MD, PhD^c^, Vivek Srivastava, MD^c^, Shakil Farid, MD, PhD^c^, Nicholas Walcot, MD^c^, Gillian Douglas, PhD^a^, Keith M Channon, MD^a,d^, Barbara Casadei, MD, PhD^a^, Charalambos Antoniades, MD, PhD^a,d^

^a^Cardiovascular Medicine Division, Radcliffe Department of Medicine, University of Oxford, UK

^b^Department of Genetics and Genomics, College of Medicine and Health Sciences, United Arab Emirates University, Al-Ain, United Arab Emirates

^c^Cardiothoracic Surgery Department, Oxford University Hospitals NHS Foundation Trust, UK

^d^Acute Multidisciplinary Imaging and Interventional Centre, Radcliffe Department of Medicine, University of Oxford, UK

**Funding:** This study was supported by a Marie Sklodowska - Curie Early Stage Researcher fellowship to M.C.C.; the CATCH ME (Characterizing Atrial fibrillation by Translating its Causes into Health Modifiers in the Elderly) consortium, grant number 633196; the British Heart Foundation (FS/16/15/32047, RG/F/21/110040 and CH/F/21/90009 to C.A.; CH/16/1/32013 to K.M.C.; CH/12/3/29609 to B.C.; Oxford BHF Centre of Research Excellence RE/18/3/34214, the Oxford NIHR Biomedical Research Centre, the National Institute for Health Research Oxford Biomedical Research Centre, and the NovoNordisk Foundation (NNF15CC0018486) to C.A.

**Disclosures:** C.A. declared past consultancy agreement with Mitsubishi Tanabe and Silence Therapeutics, past grants from Sanofi and Novo Nordisk, and honoraria from Amarin and Covance. C.A. and K.M.C. are founders, shareholders and directors of Caristo Diagnostics. C.A. is the Chair of British Atherosclerosis Society. B.C. is the past president of the European Society of Cardiology. The remaining authors have nothing to disclose.

*Drs Carena and Badi are joint first authors and contributed equally to this work.

**Address for correspondence**:

Charalambos Antoniades MD PhD FRCP FESC

BHF Chair of Cardiovascular Medicine University of Oxford

Division of Cardiovascular Medicine, L6 West Wing,

John Radcliffe Hospital, Headley Way, Oxford OX3 9DU

United Kingdom

e-mail: [antoniad@well.ox.ac.uk](mailto:antoniad@well.ox.ac.uk)

Tel: +441865228340

Twitter handle: Charis_Oxford

**Tweet:** Epicardial adipose tissue-derived miR-92a-3p is associated with improved cardiovascular outcomes and decreased myocardial oxidative stress.

**Acknowledgements:** We thank Associate Professor Christopher Toepfer and Doctor Yiangos Psaras for providing us with Human Cardiac Fibroblasts.

**CONTENT OF THE SUPPLEMENT**

| SUPPLEMENTAL METHODS | page 4 |
| --- | --- |
| REFERENCES | page 15 |
| SUPPLEMENTAL FIGURES | page 17 |
| SUPPLEMENTAL TABLE | page 27 |

**SUPPLEMENTAL METHODS**

**Study design and study population**

The study population consisted of patients undergoing cardiac surgery, all of which were recruited under the Oxford Heart Vessels and Fat (ox-HVF) program (www.oxhvf.com) at Oxford University Hospitals NHS Foundation Trust, UK. Exclusion criteria included any inflammatory, neoplastic, renal or hepatic disease.

In study arm 1 samples of epicardial adipose tissue (EAT, n=6) were collected to identify microRNAs (miRNAs) expressed and released by this tissue as described below.

In study arm 2, paired samples of EAT (from the atrioventricular groove) and right atrial appendages (RAA) were collected during surgery (n=429) to search for correlations between tissue microRNAs levels and myocardial superoxide production as well as the expression levels of their targets and downstream redox signaling.

Study arm 3 included 344 patients to search for genetic drivers of the expression levels of miRNAs detected in study arms 1-2, in both EAT and RAA. DNA was extracted from whole blood samples to perform Genome-wide genetic screening as described below.

In study arm 4, atrial myocardial specimens were collected during surgery, transferred to the lab, and used for *ex vivo* mechanistic experiments as described below.

In study arm 5 outcome data were collected from 462 patients by linking the Office for National Statistics (ONS) data with National Health Service (NHS) Digital, a nation-wide service that collects all data from the electronic patient records available in every NHS hospital in England. Events were recorded by the clinical care team, being the formal diagnosis for hospitalization or formal primary cause of death, given by the respective NHS hospitals for every hospital admission or outpatient visit. NHS digital is also connected with the UK Office for National Statistics, which offers further cross-check of the mortality data and cause of death. Patients were followed-up after surgery until the date of NHS Digital data collection or death. Follow-up time was defined as the number of days between surgery and the date of data collection (March 14, 2020) or date of death, excluding perioperative complications (within 2 weeks from surgery). Study protocols were in agreement with the Declaration of Helsinki and all participants had provided written informed consent. The demographic characteristics of these studies can be found in table 1. Supplemental Figure 1 depicts the study population, goals, and research methodologies of every study arm.

**Human tissue harvesting and processing**

Samples of EAT were harvested from the site of the right atrioventricular groove (inside the pericardial sac, attached to the heart (Supplemental Figure 2), and were used for secretome studies (see below), while snap-frozen samples were used to quantify miRNA tissue levels . RAA specimens were harvested from the cannulation site (prior to inserting the cannula in the right atrium) and collected in oxygenated (95%O_2_ /5% CO_2_) ice-cold phosphate-buffered saline (PBS). RAA specimens from patients of study arm 4 were incubated *ex vivo* with/without WNT5A 100ng/mL (645-WN/CF, R&D Systems) with/without SFRP5 300ng/mL (6266-SF, R&D Systems) prior to snap-freezing. Frozen RAA samples were stored at -80°C for gene and microRNA expression analyses and superoxide (O_2_^.-^) quantification as explained below.

**EAT secretome studies**

The EAT samples were transferred to the lab on ice within 20 minutes from harvesting and dissected into pieces, equilibrated for 1 hour at 37°C, 95% O_2_/5% CO_2_ in microvesicles-free culture Medium 199 containing fatty acid-free bovine serum albumin (1%), HEPES (25mM), gentamycin (50μg/mL), in the presence of protease inhibitor cocktail (Roche Applied Science). Microvesicle-depleted medium was obtained by ultracentrifugation at 120,000g, overnight at 37°C. After equilibration, the medium was changed (1mL of medium per 200mg tissue) for additional 4 hours incubation at 37°C, 95% O_2_/5% CO_2_. At the end of the incubation period, the supernatant collected by using a micro-sieve was processed by serial centrifugation and it was stored at –80°C until it was used for miRNA measurements.

**MicroRNAs Profiling**

***Screening phase***

EAT samples from study arm 1 were homogenized and total RNA extracted from tissue and supernatant using TriReagent (Sigma-Aldrich), according to the manufacturer’s protocol for tissues samples. RNA concentration and quality were evaluated spectrophotometrically on NanoDrop ND-1000 and on 2100 Agilent Bioanalyzer.

Total RNA was reverse transcribed using a predefined pool (Pool A) of up to 380 microRNA MegaplexTM stem-looped primers (ThermoFisher Scientific), including controls, accordingly with the manufacturer’s instruction. An additional step of pre-amplification on the supernatant samples was performed. The expression of 351 annotated human mature microRNAs and endogenous controls was profiled in the cDNA template using TaqMan human microfluidic microRNA Card A Array version 2.0 (ThermoFisher Scientific) in a 384-well format on a QuantStudio™ 7 Flex Real-Time PCR System (ThermoFisher Scientific). All microRNAs detectable in both EAT and supernatant of the 6 patients with Ct≤35 were considered to be expressed and secreted by EAT.

***Confirmation phase***

Total RNA from 206 EAT biopsies was used to prepare cDNA to detect mature microRNAs according to the miScript II RT kit manufacturer’s protocol (Qiagen) using the specific miScript HiFlex buffer. The expression of each microRNA was performed using the miScript SYBER Green PCR kit (Qiagen) in triplicate reactions with 6 ng of cDNA as template per reaction in a 384 well plate on a QuantStudio™ 7 Flex Real-Time PCR System (ThermoFisher Scientific). A series of standards was constructed by serial dilutions of a pooled cDNA sample from all patient samples; these standards were used to generate a standard curve per plate. Data were analyzed with the Pfaffl method.^1^ Plate reaction efficiency was determined based on the slope of the standard curve and the relative expression (Eff^–ΔΔCt^) of each sample was calculated by adjusting for a selected endogenous housekeeping gene expression (RNU6) and expressing the results relative to the highest standard, which served as the reference sample across plates.

**Genome-wide genetic screening**

Patients were genotyped with the genome-wide SNP array (UK Biobank Axiom Array, Affymetrix, https://www.thermofisher.com/order/catalog/product/902502). The UK Biobank Axiom 96-Array Format Automated Workflow uses the Applied Biosystems^TM^ NIMBUS^TM^ Instrument for target preparation and GeneTitan^TM^ reagent preparation to process 96 samples at a time. Sample quality control included analysis of Dish QC (DQC), call rate, heterozygosity, and sex mismatches. SNP quality control included call rate (≤97%), violation of Hardy Weinberg equilibrium (P≥10^-6^) and plate associations (P≥10^-7^). Genotypes were annotated against Axiom UKB WCSG.na35.annot.csv library that includes all the information related to each SNP and imputed using the Michigan Imputation Server (https://imputationserver.sph.umich.edu/index.html) using the version HRC r1.1, 2016, Eagle v2.3 for phasing and based on the European (EUR) Population. SNPs with Rsq > 0.7 and minor allele frequency < 0.01 were filtered. A total of 5,090,726 SNPs were considered for analysis. Genome-wide genetic screening was conducted using PLINK v1.90b3 64-bit (11 Jan 2015, Shaun Purcell & Christopher Chang). 36,543,410 variants from 344 patients in the ox-HVF cohort (study arm 3) underwent quality control. No patients were removed after filtering for a degree of missingness per individual of >5%. No variants were removed after filtering for a degree of missingness per marker of >5%. 127 variants were removed after filtering for a Hardy-Weinberg equilibrium threshold of p≤1e-08. 29,302,876 variants were then removed after filtering for a minor allelic frequency of ≥1%. Overall, 7,240,407 variants passed quality control. Quantitative trait association tests were then conducted using as quantitative phenotypes miR-92a-3p levels in EAT for “EAT-miR-92a-3p” genetic association analysis and miR-92a-3p levels in RAA for “MYO-miR-92a-3p” genetic association analysis. Estimates of the genomic inflation factor were λmedian = 1 for EAT-miR-92a-3p, and λmedian = 1.02419 for MYO-miR-92a-3p. All association values were adjusted using the Benjamini & Yekutieli (2001) step-up false discovery rate (FDR) control procedure. Following adjustment 7,227,400 variants remained in EAT-miR-92a-3p, and 7,240,000 variants remained in MYO-miR-92a-3p. Based on the adjusted association results, the top 7 single nucleotide polymorphisms (SNPs) from 3 loci in EAT-miR-92a-3p were selected to form “EAT-miR-92a-3p”, and the top 31 SNPs from 6 loci in MYO-miR-92a-3p were selected to form “MYO-miR-92a-3p”. For both clusters, dominant-coding was assumed where patients were grouped into binary categories based on whether any minor allelic variant for any of the variants in the cluster was present, compared to those that had no minor allelic variants for any of the variants in the cluster. Statistical analyses were then conducted as described.

**Cell culture experiments**

H9c2 cardiomyocytes,^2^ an embryonic cardiomyocyte-derived cell line (CRL-1446, American Type Culture Collection) were cultured in 75 cm^2^ tissue culture flasks using DMEM medium (Cat. FG0435, Millipore) supplemented with 1.5 g/L sodium bicarbonate, 10% fetal bovine serum (Cat. 0167F, Millipore), 100 U/mL penicillin and 100 μg/mL streptomycin (Cat. 15140122, ThermoFisher Scientific) at 37°C in a humidified atmosphere of 5% CO_2_. Cells were supplemented with fresh media every 3 days, and sub-cultured when 70–80% confluent in order to prevent the loss of the differentiation potential. Commercial undifferentiated H9c2 cells were then differentiated to cardiomyocytes using DMEM supplemented with 2% horse serum, 1.5 g/L sodium bicarbonate, 100 U/mL penicillin and 100 μg/mL streptomycin at 37°C in a humidified atmosphere of 5% CO_2_ for five days (Supplemental Figure 3).

TeloHAEC (CRL-4052, ATCC) are hTERT-immortalized endothelial cells isolated from the aorta of a normal 23-year-old female. They were grown in EGM-2 Endothelial Medium (CC-3162, Lonza) at 37°C in a humidified atmosphere of 5% CO_2_. Human Ventricular Cardiac Fibroblasts (CC-2904, Lonza) were a gift from Associate Professor Christopher Toepfer. They were cultured in FGM-3 Cardiac Fibroblast Growth Medium-3 (CC-4526, Lonza) at 37°C in a humidified atmosphere of 5% CO_2_ and they were used at passage 5 for superoxide generation measurements.

Cells were transfected with microRNAs mimics of the three selected microRNAs; miR-30c-5p mimic (UGUAAACAUCCUACACUCUCAGC), miR-92a-3p mimic (UAUUGCACUUGUCCCGGCCUG) and miR-193a-5p mimic (AACUGGCCCACAAAGUCCC) and a specific negative control based on a cel-miR-67 (*C. elegans*) mature sequence (UCACAACCUCCUAGAAAGAGUAGA) (Exiqon). TeloHAEC and human ventricular cardiac fibroblasts were seeded at a density of 20,833 and 10,417 cells/cm^2^, respectively, and transfected after 24 hours; differentiated H9c2 were transfected at day 3 of differentiation. Darmaphect 1 (Horizon Discovery) was used for transfections following the manufacturer’s instructions, for 48 hours at a final concentration of 25nM. To assess Akt involvement in miR-92a-3p-mediated effects differentiated H9c2 were treated for one hour with perifosine 10µM (Cat. 14240, Cell Signaling). Treated cells were harvested for Western blot analysis, Rac1 activation and membrane translocation assessments, and for O_2_^.-^ generation measurements.

FLAG-tagged Wnt5a was stably overexpressed in H9c2 cells after selection with hygromycin of cells transfected with pCMV-mWNT5A-FLAG vector (Sino Biologicals) using Fugene (Promega). Expression of the transgene was confirmed by immunofluorescence using mouse anti-FLAG M2 antibody (F3165, Sigma-Aldrich). Differentiated H9c2 cells were also incubated with/without WNT5A 100ng/mL and/or SFRP5 300ng/mL for 30min in a 5% CO_2_ incubator at 37°C, then lysed for further analyses as described below. For the Rac1 inhibition experiment, differentiated H9c2 cells were pre-incubated with NSC23766 100μM (R&D systems, catalogue number 2161) for 15min prior to WNT5A treatment and O_2_^.-^ generation assessment.

**Animal Experiments**

A doxycycline-inducible Wnt5a knock-in mouse model was used to determine the *in vivo* effects of Wnt5a on myocardial NADPH oxidase activity. C57BL/6, FVB/N Tg(tetO- Wnt5a)17Rva/J (TetO-Wnt5a^+^) and C57BL/6, FVB CAG-rtTA (rtTA^+^) mouse lines have been previously described.^3^ 2mg/mL doxycycline hyclate (J60579, Alfa Aesar) was administered to male and female, 8-14 week old double transgenic tetO-Wnt5a^+^/rtTA^+^ and their littermate tetO-Wnt5a^–^/rtTA^+^ controls via the drinking water, containing 2.5% sucrose overnight to induce Wnt5a expression. Considerable weight loss (up to ~15% of body weight) was observed after three days of doxycycline treatment, therefore this mouse model was not suitable for long-term experiments as per local committee ethics. Overnight doxycycline treatment induced minor weight loss and did not compromise the welfare of the animals. DNA extracted from experimental animal ear notches was used for genotyping with the following PCR primers:

C57BL/6, FVB CAG-rtTA

CCM 5’- CGAAACTCTGGTTGACATG - 3’

CTG 5’- ATGCCCTGGCTCACAAATAC - 3’

CWT 5’- TGCCTATCATGTTGTCAAA – 3’

C57BL/6, FVB/N Tg(tetO- Wnt5a)17Rva/J

17815 5’- ACAAAGACGATGACGACAAGC – 3’

17816 5’- CGCACCTTCTCCAATGTACTG – 3’

oIMR7338 5’- CTAGGCCACAGAATTGAAAGATCT – 3’

oIMR7339 5’ – GTAGGTGGAAATTCTAGCATCATCC – 3’

Mice were housed in a specific pathogen-free environment, in Tecniplast Sealsafe IVC cages (floor area 542 cm^2^) with a maximum of 5 other mice. Mice were kept in a 12 h light/dark cycle and in controlled temperatures (20–22°C) and fed normal chow and water *ad libitum*. Genotyping results were not made available to personnel performing phenotyping and data collection. Myocardial superoxide measurements were thus carried out by personnel blinded to genotype. No samples or animals were excluded from data analysis.

**Superoxide production measurements**

Myocardial O_2_^.-^ production was measured in the human atrial myocardium or murine hearts using lucigenin (5μmol/L)-enhanced chemiluminescence, as we have previously described.^4,5^ Myocardial tissue was homogenized in ice-cold Krebs HEPES Buffer (pH 7.35) in the presence of protease inhibitor (Roche Applied Science) using a pre-cooled Polytron homogenizer. Protein concentration was quantified and unified to 0.2 mg before the experiment.

The contribution of NADPH oxidase activity to myocardial O_2_^.-^ production was quantified in the presence of NADPH at 100μmol/L. Vas2870 (400 μmol/L; Sigma-Aldrich, a specific pan-NADPH oxidase inhibitor) was used to obtain the Vas2870-inhibitable O_2_^.-^ signal, which constitutes a more specific index of NADPH oxidase activity as previously reported ^4^.

O_2_^.-^ was also quantified in cell lysates. Following *in vitro* transfections and/or incubations, cells were scraped in ice-cold Krebs HEPES Buffer (pH=7.35) in the presence of protease inhibitor (Roche Applied Science). The lysates were sonicated and processed similarly to tissue homogenates as described above in order to quantify the various O_2_^.-^ sources.

**RNA isolation and quantitative real time-polymerase chain reaction (PCR)**

***RNA isolation***

Total RNA was isolated by phenol:chloroform (1:5 ratio) separation followed by magnetic beads-based RNA purification on a KingFischer magnetic particle processor (ThermoFischer Scientific) by using the MagMAX mirVana total RNA isolation kit (A27828, ThermoFischer Scientific). RNA concentration was evaluated spectrophotometrically on NanoDrop ND-1000.

***Reverse transcription***

RNA was reverse-transcribed to cDNA by using SuperScript VILO mastermix (ThermoFischer Scientific) following the Manufacturer’s instructions and extending the cDNA synthesis step to two hours at 60°C on a Veriti thermal cycler (ABI).

***Quantitative real-time PCR***

Quantitative real-time PCR was performed by TaqMan chemistry, using the standard universal TaqMan protocol as indicated by the Manufacturer, on a QuantStudio 7 flex real-time PCR system (ThermoFischer Scientific). All samples were run in duplicates using 5 ng of cDNA as starting mass, and data were analyzed with the Pfaffl method ^1^. PGK1 was used as housekeeping gene for human RAA. The IDs of the TaqMan probes used are: PGK1: Hs00943178_g1; WNT5A: Hs00998537_m1; SFRP5: Hs00169366_m1; PHLPP2: Hs00982295_m1; FZD2: Hs00361432_s1; FZD5: Hs00258278_s1; ROR1: Hs00938677_m1; ROR2: Hs00896176_m1; RYK: Hs00243196_m1; Wnt5a: Mm00437347_m1; 18S RNA: Mm04277571_s1.

**Rac1 activation**

Rac1 activation was assessed using an active Rac1 detection kit (Cell Signaling). Briefly, H9c2 cells, human RAA samples or mouse heart tissue were lysed or homogenized as appropriate, using the lysis buffer provided by the kit, supplemented with protease and phosphatase inhibitors. Lysates (500 µg) were then incubated with glutathione resin and GST-PAK1-PBD according to the manufacturer’s specific instructions (Cell Signaling). Western blot analysis was performed as described below; Rac1 activation is presented as the ratio of guanosine triphosphatase (GTP)-Rac1/total Rac1.

**Rac1 membrane translocation**

Membrane translocation of Rac1 was estimated by differential centrifugation of cell lysates or tissue homogenates to isolate membrane proteins as previously described.^3^ Briefly, debris was removed by centrifugation of the homogenates at 2,800 g at 4°C for 20min, and protein content of supernatants was evaluated by the Pierce BCA protein assay kit. 500mg of total protein were adjusted to 200μL for all samples, added into ultracentrifugation tubes and ultra-centrifuged at 100,000 g for 60 min at 4°C to separate cytosolic from membrane proteins. Following removal of supernatants containing the cytosolic proteins, pellets were resuspended in 35μL of lysis buffer containing 1% Triton and left for 20 min on ice. Membrane-translocated Rac1 was determined by Western immunoblotting as described below.

**Western blotting**

Cells were washed in cold PBS and lysed in RIPA buffer (Cell Signaling) containing proteases and phosphatases inhibitors (Roche). Human RAA and mouse heart specimens were homogenized in ice-cold RIPA buffer (Cell Signaling) supplemented with proteases and phosphatases inhibitors (Roche) by using a Polytron homogenizer. Protein extract concentrations were determined using the BCA assay (Pierce), with albumin as standard.

20μg of denatured protein extracts were resolved by sodium dodecyl sulphate-polyacrylamide gel electrophoresis (SDS-PAGE) on 4-20% polyacrylamide gradient gel, and the proteins were then transferred to nitrocellulose membranes (Amersham). The primary antibodies used were: anti-phospho-Akt (Ser473) (#4060, Cell Signaling, 1:1000), anti-Akt (pan) (#4691, Cell Signaling, 1:1000), anti-PHLPP2 (NB100-1812, Novus Biological, 1:2000), anti-PTEN (#9552, Cell Signaling, 1:1000), anti-Rac1 (#8631, Cell Signaling, 1:1,000), anti-Wnt5a (#2530, Cell Signaling, 1:1,000), and anti-GAPDH HRP-conjugated (G9295, Sigma-Aldrich 1:20,000). HRP-conjugated anti-rabbit (A9169, Sigma-Aldrich, 1:10,000) and HRP-conjugated anti-mouse (A9044, Sigma-Aldrich, 1:15,000) were used as secondary antibodies as appropriate. For detection of immuno-reactive bands, ECL select Western Blotting Detection Reagent (Amersham) was used. Bands were quantified using Image Lab Bio-rad software integrated density analysis.

**Statistical analysis**

Continuous variables were tested for normal distribution using the Kolmogorov-Smirnov test. Non-normally distributed variables are presented as median [25th-75th percentile] and whiskers (from 10th to 90th percentile) for n≥30. In experiments with small sample size (n<30), variables are presented as scatterplots with median value. Correlations between two continuous variables were evaluated by Pearson (for normally distributed variables) or Spearman (for non-normally distributed variables) coefficients as appropriate. Comparisons of continuous variables between two groups were performed using unpaired t test or Mann-Whitney U-test as appropriate, while comparisons between 3 or more groups were performed using one-way ANOVA or Kruskal-Wallis followed by Dunn’s test for multiple comparisons. Paired comparisons were performed using paired t test or Wilcoxon signed-rank test as appropriate.

Mechanistic experiments were performed in a paired design in human samples *ex vivo* and in H9c2 cells *in vitro*. Results were analyzed by Wilcoxon signed-rank tests or paired t-tests between control-intervention pairs followed by Bonferroni post hoc correction for multiple comparisons.

To test the association of levels of miR-92a-3p in EAT or *WNT5A/SFRP5* in the myocardium with the composite outcome of cardiac mortality, non-fatal myocardial infarction (MI) and non-fatal stroke, we created dichotomous categorical variables by splitting the population of study arm 5 into two groups using as cut-offs the top tertile for EAT miR-92a-3p levels and myocardial *WNT5A/SFRP5* levels. The effect of miR-92a-3p levels in EAT or myocardial *WNT5A/SFRP5* on the composite outcome was then examined by multivariate Cox regression survival analysis after adjusting for age, sex, hypertension, BMI and diabetes.

All statistical tests were two-tailed and were performed using SPSS version 20.0. P<0.05 was considered statistically significant.

**REFERENCES**

1. Pfaffl MW. A new mathematical model for relative quantification in real-time RT–PCR. Nucleic Acids Res 2001;29:e45-e45.

2. Hescheler J, Meyer R, Plant S, Krautwurst D, Rosenthal W, Schultz G. Morphological, biochemical, and electrophysiological characterization of a clonal cell (H9c2) line from rat heart. Circ Res 1991;69:1476-1486.

3. Akoumianakis I, Sanna F, Margaritis M et al. Adipose tissue–derived WNT5A regulates vascular redox signaling in obesity via USP17/RAC1-mediated activation of NADPH oxidases. Sci Transl Med 2019;11:eaav5055.

4. Antonopoulos AS, Margaritis M, Verheule S et al. Mutual regulation of epicardial adipose tissue and myocardial redox state by PPAR-γ/adiponectin signalling. Circ Res 2016;118:842-855.

5. Reilly SN, Jayaram R, Nahar K et al. Atrial sources of reactive oxygen species vary with the duration and substrate of atrial fibrillation: implications for the antiarrhythmic effect of statins. Circulation 2011;124:1107-1117.

**
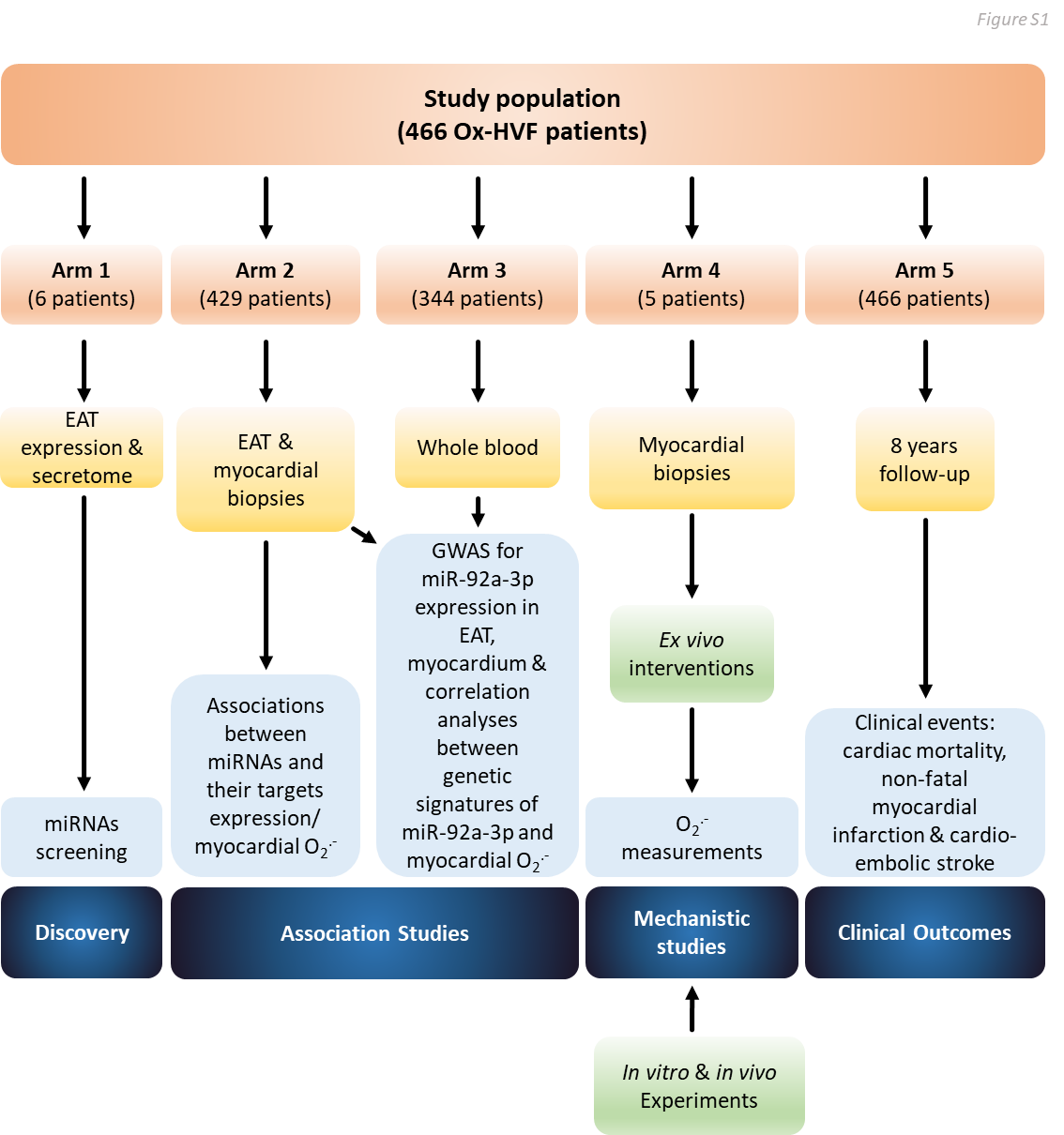
**

**Supplemental Figure 1**. **Study Design.** Schematic diagram of the study population, goals, and research methodologies. EAT: epicardial adipose tissue; GWAS: Genome-Wide Association Studies; O_2_^.-^: superoxide; Ox-HVF: Oxford Heart Vessels and Fat cohort; RAA: right atrial appendages.


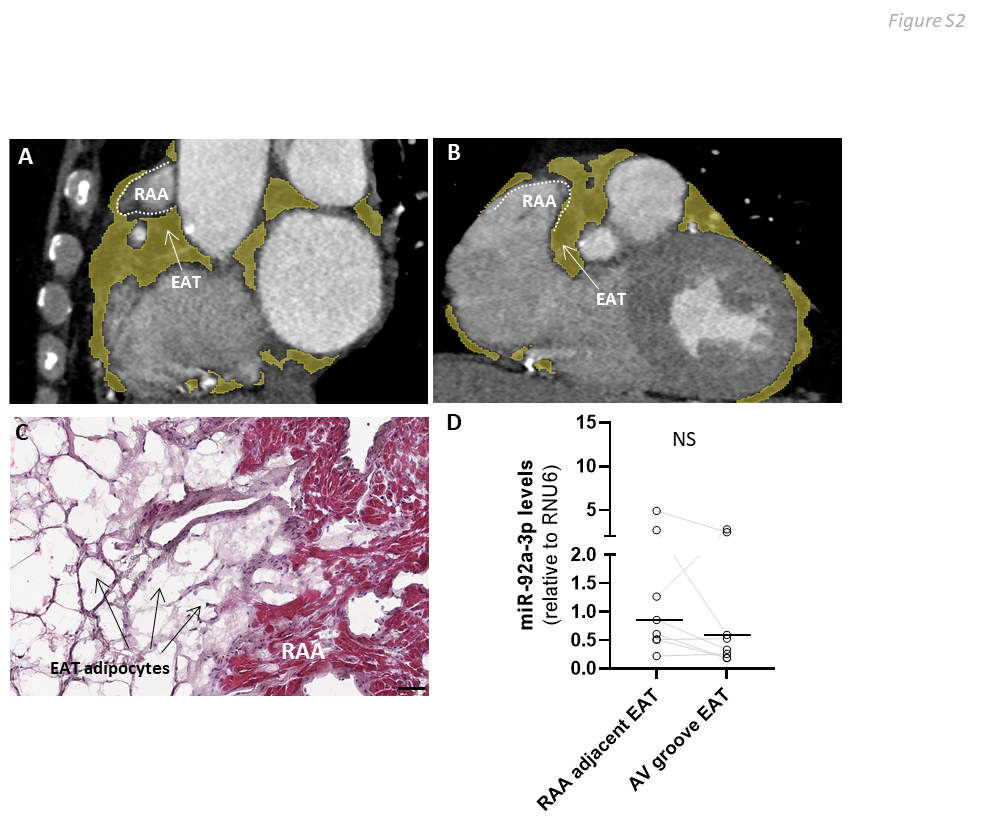


**Supplemental Figure 2**. **Relationship between epicardial adipose tissue (EAT) and right atrial appendage (RAA).** (**A-B**) Coronary Computed Tomography Angiography (CCTA) image showing that the same EAT (coloured in yellow) depot extends from the atrioventricular groove to the RAA. (**C**) Haematoxylin and eosin staining that shows the anatomical proximity between adipose tissue and the right atrial appendage (RAA). The sample was embedded in OCT and a 20 µm section was stained and acquired with a Leica Biosystems Aperio CS2 scanner at 40x magnification. The image displays a 17x magnification. Scalebar: 60 µm. (**D**) miR-92a-3p levels in paired EAT samples adjacent to the RAA (within 0.5cm) vs from the right atrioventricular (AV) groove. NS, not significant using Wilcoxon paired rank test.


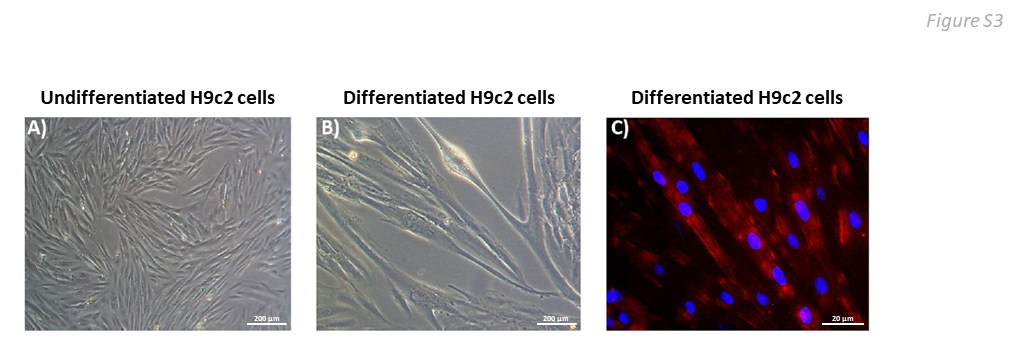


**Supplemental Figure 3**. **Characterization of the differentiated cardiomyocytes *in vitro* model.** Bright-field representative images of undifferentiated rat H9c2 cardiomyoblasts (**A**) and differentiated cardiomyocytes obtained after growing confluent cardiomyoblasts for 5 days in differentiation medium (**B**, see Materials and Methods). (**C**) Representative image of staining of differentiated cardiomyocytes with the fluorescent dyes DAPI (for nuclei, in blue) and Phalloidin-Texas red (for F-actin, in red).


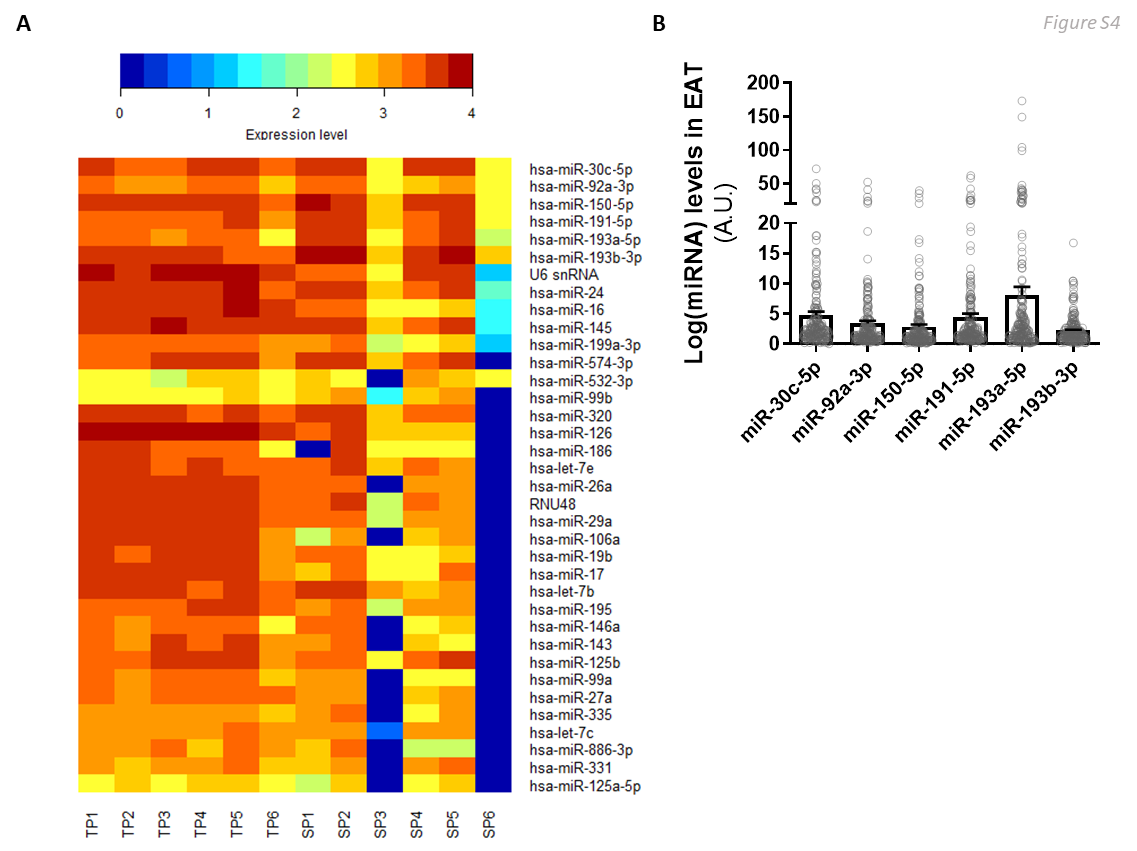


**Supplemental Figure 4**. **MicroRNAs levels profile in human EAT and corresponding supernatants.** (**A**) Hierarchical clustering of differentially expressed microRNAs in EAT and corresponding supernatants obtained from 6 patients from the Oxford Heart Vessels and Fat (Ox-HVF) cohort that had undergone cardiac surgery. (**B**) Validation of the expression in EAT in n=206 patients of the six microRNAs that were found expressed and released by EAT from all the six patients in the discovery phase. TP1,2,3,4,5,6: EAT of patient 1,2,3,4,5,6; SP1,2,3,4,5,6: supernatant from EAT of patient 1,2,3,4,5,6.

**
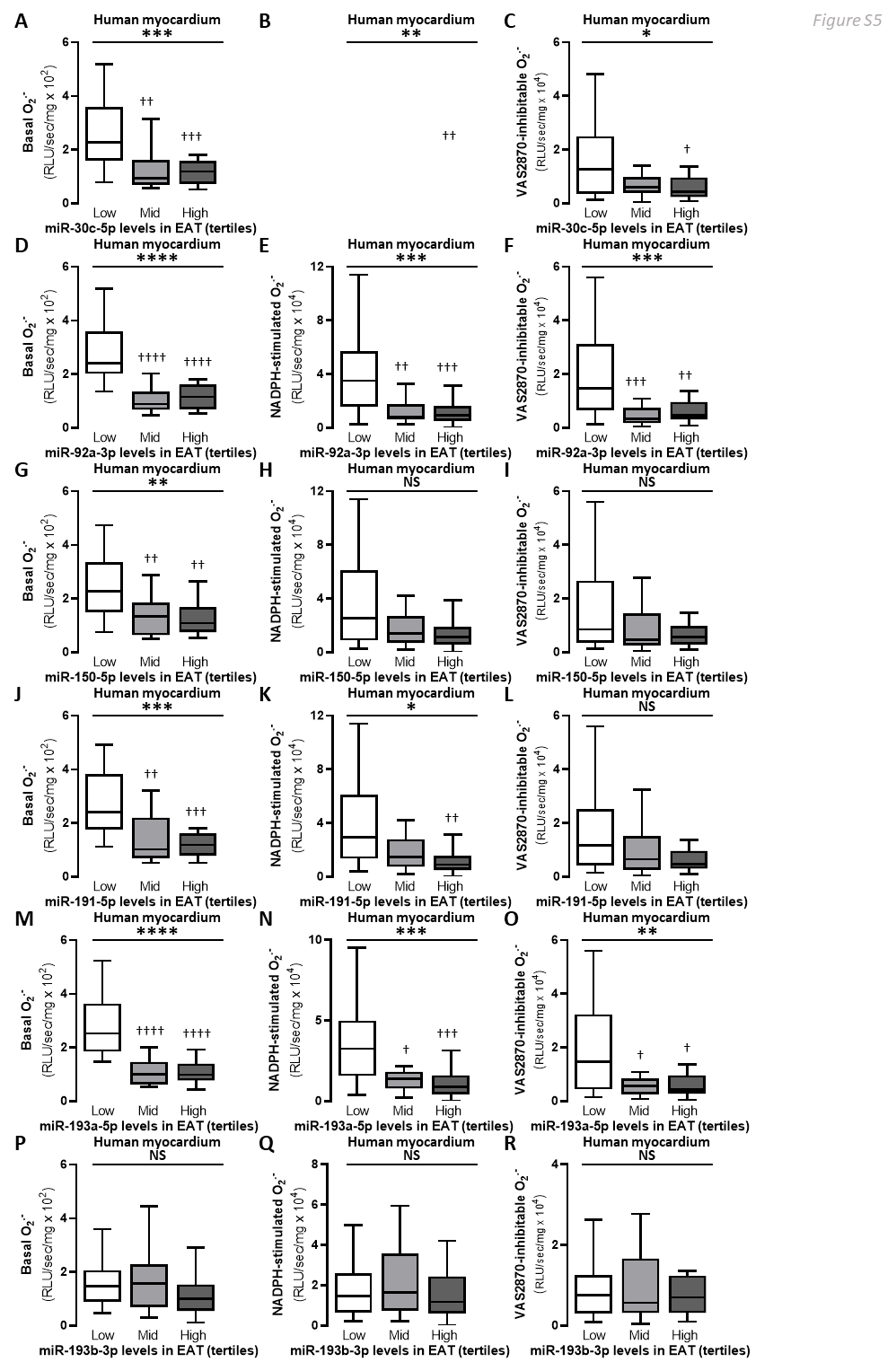
Supplemental Figure 5**. **miRNAs levels in EAT and superoxide generation in paired myocardium.** Basal, NADPH-stimulated and Vas2870-inhibtable superoxide (O_2_^.-^) production in human myocardium according to tertiles of miR-30c-5p (**A** through **C**), miR-92a-3p (**D** through **F**), miR-150-5p (**G** through **I**), miR-191-5p (**J** through **L**), miR-193a-5p (**M** through **O**), and miR-193b-3p (**P** through **R**) levels in EAT. Data are presented as median [25th-75th percentile] (n=56). *P<0.05; **P<0.01; ***P <0.001; ****P<0.0001 by Kruskal-Wallis test. † P<0.05; ††Adjusted P<0.01; †††Adjusted P <0.001; ††††Adjusted P<0.0001 vs low tertile by Dunn’s test adjusted for multiple tests. NS, not significant. RLU, relative light units.


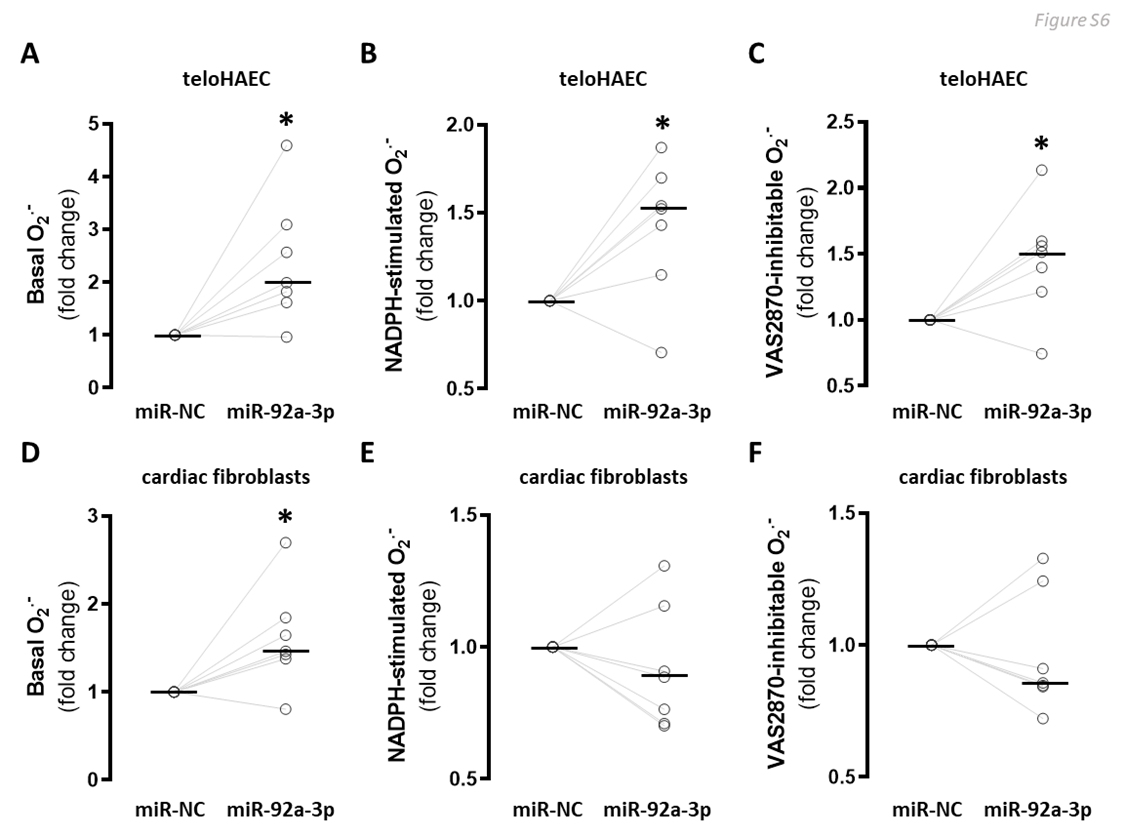


**Supplemental Figure 6.** Effects of **miR-92a-3p on redox state of endothelial cells and cardiac fibroblasts.** (A-F) Basal, NADPH-stimulated and Vas2870-inhibtable O_2_^.-^ generation in teloHAEC and human cardiac fibroblasts transfected with a miRNA mimic negative control (miR-NC) or miR-92a-3p (n=7). Lines represent the median value. *P<0.05 vs control by Wilcoxon signed rank test.


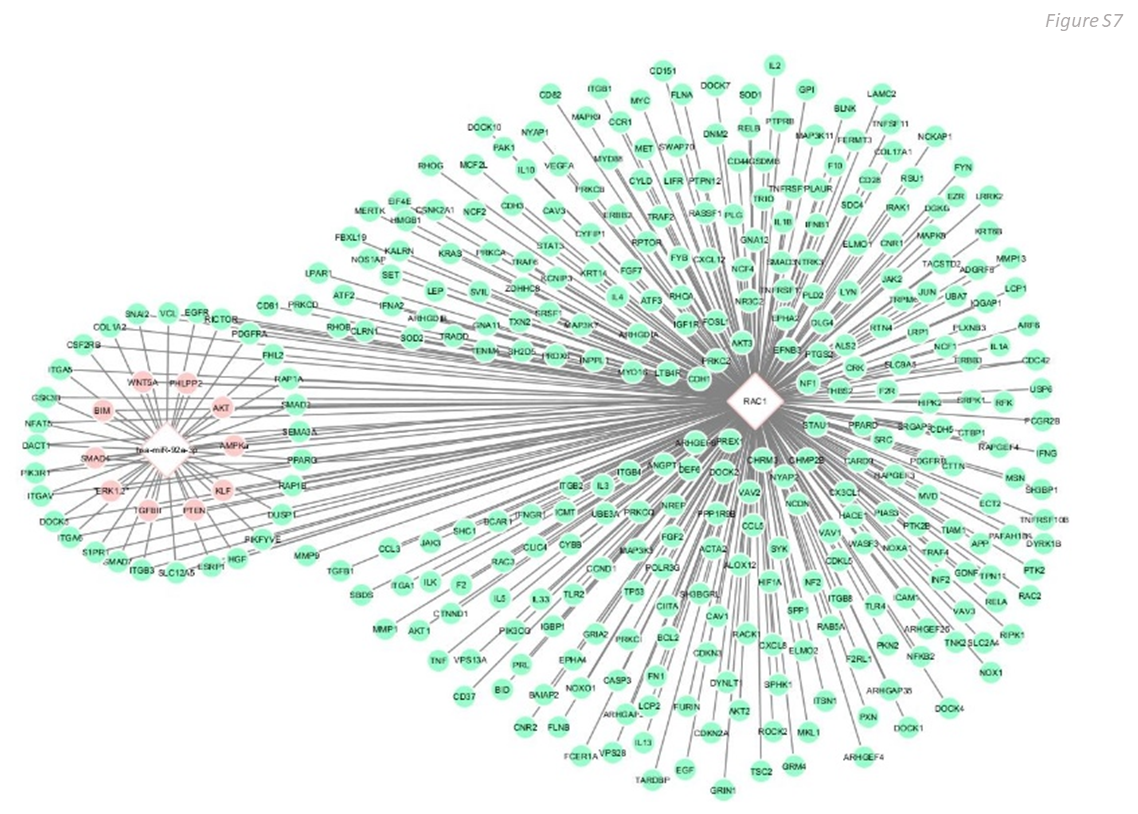


**Supplemental Figure 7**. **Assessment of the mechanisms through which miR-92a-3p may affect Rac1.** The protein-protein interactions between Rac1 and several *in silico* predicted targets of miR-92a-3p are visualized by Cytoscape (https://cytoscape.org/). Each node represents a protein and each line refers to an interaction. The pink circles indicate miR-92a-3p target proteins that are involved in oxidative stress.


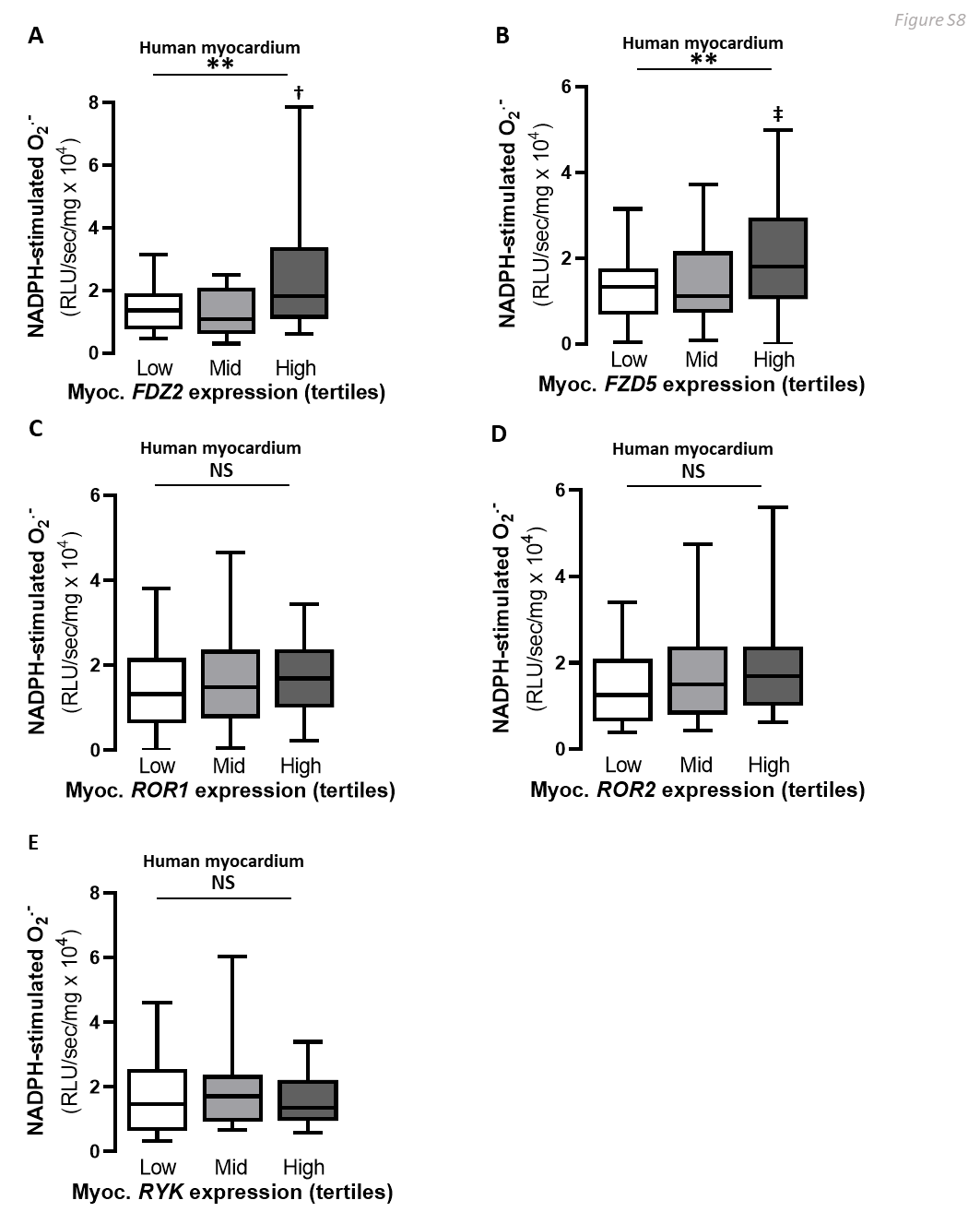


**Supplemental Figure 8**. **NOX activity and Wnt receptor expression in the human myocardium.** NADPH-stimulated superoxide (O_2_^.-^) production in human myocardium according to tertiles of levels of expression of different WNT5A receptors (Frizzled class receptor 2, FZD2; Frizzled class receptor 5, FZD5; receptor tyrosine kinase like orphan receptors 1 & 2, ROR1-2; and receptor like tyrosine kinase, RYK) in human myocardium. Data are presented as median [25th-75th percentile] (n=181). **P<0.01 by Kruskal-Wallis test. †P<0.05 vs low tertile by Dunn’s test followed by correction for multiple tests. NS, not significant.

**
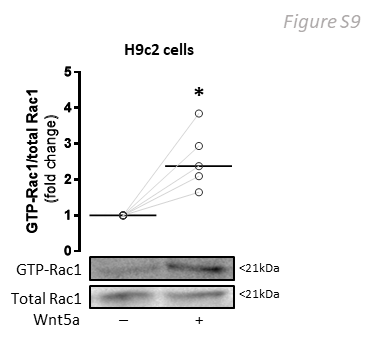
**

**Supplemental Figure 9**. **Wnt5a overexpression in H9c2 cells increases Rac1 GTP-activation.** Fold change of activated Rac1 in H9c2 cells overexpressing or not FLAG-tagged Wnt5a (n=5). Lines represent the median values. *P<0.05 vs control by Wilcoxon signed rank test.

**Supplemental Table 1**: Comparisons of clinical characteristics of patients stratified by miR-92a-3p levels in epicardial adipose tissue (EAT).

| **EAT miR-92a-3p (tertiles)** | **Low** | **Medium** | **High** | **p-value** |
| --- | --- | --- | --- | --- |
| Age (years) | 67±1.3 | 64±1.5 | 68±1.2 | 0.101 |
| Male sex (%) | 73.9 | 73.5 | 87.0 | 0.095 |
| Hypertension (%) | 73.4 | 79.4 | 79.7 | 0.195 |
| Hypercholesterolaemia (%) | 68.1 | 69.1 | 78.3 | 0.037 |
| Type 2 Diabetes Mellitus (%) | 26.1 | 19.1 | 24.6 | 0.596 |
| Smoking (active/ex) (%) | 69.6 | 60.3 | 69.6 | 0.119 |
| BMI (Kg/m^2^) | 28.84±0.5 | 28.01±0.6 | 29.26±0.6 | 0.495 |
| Triglycerides (mM) | 1.42±0.10 | 1.42±0.18 | 1.44±0.10 | 0.345 |
| HDL-Cholesterol (mM) | 0.92±0.03 | 0.96±0.03 | 0.86±0.02 | 0.095 |
| LDL-Cholesterol (mM) | 2.01±0.11 | 1.93±0.10 | 1.91±0.09 | 0.795 |
| **Medications (%)** |  |  |  |  |
| ACEi | 43.5 | 50.0 | 50.7 | 0.644 |
| Antiplatelet | 71.0 | 76.5 | 88.4 | 0.039 |
| ARBs | 15.4 | 19.1 | 11.6 | 0.475 |
| Beta blockers | 52.2 | 61.8 | 71.0 | 0.075 |
| Statins | 75.4 | 77.9 | 82.6 | 0.575 |
| CCBs | 24.6 | 29.4 | 27.5 | 0.818 |
| Insulin | 2.9 | 5.9 | 11.6 | 0.119 |
| Oral antidiabetics | 21.7 | 11.8 | 15.9 | 0.287 |
| Metformin | 20.3 | 8.8 | 14.5 | 0.164 |
| Sulfonylurea | 13.0 | 7.4 | 7.2 | 0.405 |
| DPP4-Inhibitors | 0 | 2.9 | 0 | 0.129 |
| GLP1-Analogues | 1.4 | 0 | 0 | 0.369 |

Values are presented as % if not otherwise stated, and averaged values as presented as mean±SEM. Abbreviations: (ACEi) Angiotensin converting enzyme inhibitors, (ARBs) Angiotensin receptor blockers, (BMI) Body mass index, (CCBs) Calcium channels blockers, (DPP4) Dipeptidyl Peptidase-4, (GLP1) Glucagon-Like Peptide 1, (HDL) High Density Lipoprotein, (hsCRP) high-sensitivity C-Reactive Protein, (LDL) Low Density Lipoprotein.
